# Defective *APETALA2* Genes Lead to Sepal Modification in *Brassica* Crops

**DOI:** 10.3389/fpls.2018.00367

**Published:** 2018-03-20

**Authors:** Yanfeng Zhang, Shuhua Huang, Xuefang Wang, Jianwei Liu, Xupeng Guo, Jianxin Mu, Jianhua Tian, Xiaofeng Wang

**Affiliations:** ^1^Hybrid Rapeseed Research Center of Shaanxi Province, Yangling, China; ^2^State Key Laboratory of Crop Stress Biology for Arid Areas, College of Horticulture, Northwest A&F University, Yangling, China

**Keywords:** *APETALA2* (*AP2*), carpeloid sepal, *Brassica*, flower development, oilseed rape, organ modification

## Abstract

Many vegetable and oilseed crops belong to *Brassica* species. The seed production of these crops is hampered often by abnormal floral organs, especially under the conditions of abiotic conditions. However, the molecular reasons for these abnormal floral organs remains poorly understood. Here, we report a novel pistil-like flower mutant of *B. rapa*. In the flower of this mutant, the four sepals are modified to one merged carpel that look like a ring in the sepal positions, enveloping some abnormal stamens and a pistil, and resulting in poor seed production. This novel mutant is named *sepal-carpel modification* (*scm*). DNA sequencing showed that the *BrAP2a* gene, the ortholog of *Arabidopsis APETALA2* (*AP2*) that specifies sepal identity, losses the function of in *scm* mutant due to a 119-bp repeated sequence insertion that resulted in an early transcription termination. *BrAP2b*, the paralog of *BrAP2a* featured two single-nucleotide substitutions that cause a single amino acid substitution in the highly conserved acidic serine-rich transcriptional activation domain. Each of the two *BrAP2* genes rescues the sepal defective phenotype of the *ap2-5* mutant of *Arabidopsis*. Furthermore, the knockout mutation of the corresponding *BnAP2* genes of oilseed rape (*B. napus*) by CRISPR/Cas9-mediated genome editing system resulted in *scm*-like phenotype. These results suggest that *BrAP2* gene plays a key role in sepal modification. Our finding provides an insight into molecular mechanism underlying morphological modification of floral organs and is useful for genetic manipulation of flower modification and improvement of seed production of *Brassica* crops.

## Introduction

Eudicot flowers are composed of four distinct floral organs, sepals, petals, stamens, and carpels, which are arranged in four concentric whorls from the outer to the inner layer of the flower. Genetic analysis of floral homeotic mutants in *Arabidopsis thaliana* and *Antirrhinum majus* led to the development of the classic ABC model of floral organ identity (Bowman et al., 1991; Coen and Meyerowitz, 1991). This model proposes three classes of floral homeotic genes that act alone or cooperate with each other to specify the four floral organs: class A specifies the sepals, A and B specify the petals, B and C specify the stamens, and C specifies the carpels. A mutation in the A-function gene generates a homeotic transition of sepals to carpels and petals to stamens. A B-functional gene mutation gives rise to a homeotic transformation of petals to sepals and stamens to carpels. A C-functional gene mutation leads to a homeotic translation of stamens to petals and carpels to sepals (Meyerowitz et al., 1991; Weigel and Meyerowitz, 1994).

In *Arabidopsis*, transcription factor *APETALA1* (*AP1*) and *APETALA2* (*AP2*) are A-functional genes, *APETALA3* (*AP3*) and *PISTILLATA* (*PI*) are B-functional genes, while *AGAMOUS* (*AG*) is a C-functional gene (Yanofsky et al., 1990; Bowman et al., 1993; Jofuku et al., 1994; Krizek and Meyerowitz, 1996). In addition, it has been shown that *SEPALLATA* orthologs (*SEP1*–*SEP4*) are also required to specify floral organ identity (Pelaz et al., 2000). These genes are defined as E-function genes, and their discovery led to the proposal of the ABCE model (Jack, 2001). All of these well-characterized genes, except for *AP2*, are members of the MADS-box family encoding MIKC-type MADS-box transcription factors (Saedler et al., 2001; Theissen, 2001; Kaufmann et al., 2005). These encoded proteins constitute four different tetrameric complexes that specify the various corresponding floral organs. Accordingly, the AP1, AP1, SEP, and SEP tetramer determines the sepal development; AP1, AP3, PI, and SEP complex determines the petal development; AP3, PI, AG, and SEP determines the stamen development; while the AG, AG, SEP, and SEP tetramer determines the development of carpel (Yanofsky et al., 1990; Honma and Goto, 2001; Pelaz et al., 2001; Melzer and Theißen, 2009). This protein complexes-based model of floral organ specification is referred to as the quartet model, and is an improved successor of the ABCE theory (Theissen and Saedler, 2001). So far, the ABC model and its improved, more detailed versions have proven to be useful frameworks for interpreting floral development in a variety of species. However, there is little evidence to support the fact that the A-function genes specify the development of sepals and petals in non-*Arabidopsis* plant species.

In *Arabidopsis*, both A-functional genes *AP1* and *AP2* are required for sepal and petal development (Kunst et al., 1989; Irish and Sussex, 1990; Drews et al., 1991; Bowman et al., 1993; Gustafson-Brown et al., 1994). Indeed, *Arabidopsis* is the only species in which floral organ identity defects have been linked to A-functional genes (Davies et al., 1999; Litt, 2007; Causier et al., 2010). Interestingly, the *SQUAMOSA* (*SQUA*) gene, an *AP1* ortholog in *Antirrhinum majus*, plays a key role in determining floral meristem identity, but does not affect the specification of sepals and petals in this species (Huijser et al., 1992). Meanwhile, *AP2* was originally identified in *Arabidopsis* as a floral homeotic gene involved both in the A-functional roles of specifying organ identity and in restricted C-functional activities. Loss of *AP2* function has previously been shown to cause a homeotic transition of sepals to carpels, and petals to stamens (Drews et al., 1991; Bowman et al., 1993; Causier et al., 2010). Further investigations revealed that the *AP2* gene has numerous additional functions in the regulation of flowering time (Aukerman and Sakai, 2003), maintenance of the shoot apical meristem (Wurschum et al., 2006), and the determination of seed and fruit development (Maes et al., 2001; Jofuku et al., 2005; Ohto et al., 2005; Ripoll et al., 2011; Zhou et al., 2012). However, the exact role of the *AP2* gene in flower development, especially in sepal and petal specification, has not been determined. Therefore, exploring of novel A-functional homeotic mutants in non-*Arabidopsis* species is currently critical to verify the functions of the *AP1* and *AP2* genes in sepal and petal specification, which could contribute new details to complement the classic ABC model of floral organ identity.

Many vegetable and oilseed crops belong to *Brassica* species. The seed production of these crops is hampered often by abnormal floral organs, especially under conditions of abiotic stress (Zhou et al., 2008; Huang et al., 2010). Similar to *Arabidopsis*, both *B. rapa* and *B. napus* are members of the Cruciferae family. *B. napus* is an allodiploid species derived from spontaneous hybridization between *B. rapa* and *B. oleracea*, and possesses the complete diploid chromosome sets of the parental genomes (Parkin et al., 1995; Snowdon et al., 2002). In our previous study, we recovered a novel pistil-like flower mutant with *sepal-to-carpel modification* (*scm*) in an F_3_ selfing line of an interspecific cross between *B. napus* and *B. rapa*, which prompted us to further explore the molecular mechanism underlying its phenotype. Here we report that the *scm* mutant was apetalous resulting in poor seed production. Further studies indicated that the mutations in the two *AP2* orthologs in *B. rapa* (*BrAP2* genes) genes of *B. rapa* (Song et al., 2013) were the fundamental causes of the defects.

## Materials and Methods

### Plant Materials

The *scm* and *scm2* from the same F_3_ selfing line Nc116 were derived by an interspecific cross between *B. napus* and *B. rapa*. Both wild-type *B. rapa* (WTr) and *B. napus* (WTn) were used as controls. The *AP2* weak mutant *ap2-5* of *Arabidopsis thaliana* (stock name CS6239, purchased from the website^1^) with a mild sepal-to-carpel and petal-to-stamen phenotype was used as transgenic receptor material.

### RNA Extraction and Gene Expression Assays

Total RNA was extracted from young inflorescence at the budding stage using RNAiso Reagent (TaKaRa, Dalian, China). The first strand cDNA was synthesized using the Prime Script RT-PCR kit (TaKaRa, Dalian, China). Primers specific for the *AP1*, *AP2*, *AP3*, *PI*, *AG*, and *SEP3* genes were designed based on sequences retrieved from the *Brassica* database^2^ and the NCBI nucleotide and EST databases (details of primers are given in Supplementary Table 1). The expression of these genes was determined using semi-quantitative RT-PCR. The expression levels of the target genes were normalized to the internal control, the cytosolic *18S rRNA* gene. The PCR products were separated on a 1% agarose gel, EtBr stained, purified using the Omega DNA Gel Extraction Kit (Omega Bio-Tek), and cloned into the pGEM-T Easy Vector (Promega, Madison, WI, United States) for sequencing (Beijing Sunbiotech Co., Ltd.).

### Generation of Transgenic *Arabidopsis* Lines of the Two *B. rapa AP2* Genes

To produce GFP-tagged *AP2* genes from *B. rapa* (*Br*) and *Arabidopsis* (*At*), the coding sequences (CDSs) without the stop codon of the *BrAP2a*, *BrAP2b*, and *AtAP2* (control) were PCR-amplified using the following primer sets: BAP2-F+*Sac* I/BAP2-R2a+*Xba* I, BAP2-F+*Sac* I/BAP2-R2b+*Xba* I, and AtAP2-F+*Kpn* I/AtAP2-R+*Xba* I, respectively (Supplementary Table 1). The amplicons were checked by sequencing, and the full-length CDSs were then cloned into the *Sac* I and *Xba* I sites of Cam-35S-GFP-containing vectors, resulting in the C-terminal fusion to GFP.

The resultant Cam-35S-*BrAP2a*-GFP, Cam-35S-*BrAP2b*-GFP, and Cam-35S-*AtAP2*-GFP fusion constructs were transformed into the *Agrobacterium tumefaciens* strain GV3101 using electroporation and subsequently infiltrated into *Arabidopsis ap2-5* and Col-0 plants are using the floral dip method (Clough and Bent, 1998). T1 seeds from the infiltrated plants were selected and plated on 1/2 MS medium containing 33 mg/L hygromycin B.

### Western Blot Assay

To examine the expression of the *AtAP2-GFP*, *BrAP2a-GFP*, and *BrAP2b-GFP* genes in independent transgenic lines, 50 mg of young leaves were ground in liquid N_2_ and treated with 200 μL 1×SDS loading buffer without Bromophenol Blue (50 mM pH 6.8 Tris–HCl, 5% β-mercaptoethanol, 10% Glycerol, and 2% SDS), followed by further grinding. The homogenate was boiled for 5 min and centrifuged for 5 min at 10,000 *g*. The supernatant was collected, and its protein concentration was determined using a Bradford assay. Samples containing 50 μg of protein were subjected to 10% SDS–PAGE and transferred to a PVDF membrane (Roche, Mannheim, Germany). Immunoblot analysis was performed using an anti-GFP (1:2000) monoclonal antibody (Beijing TransGen Biotech Co., Ltd.). The chemiluminescent HRP substrate (Millipore) was then added, incubated for 5 min, and the blots were imaged using Image Lab Software (Bio-Rad, Hercules, CA, United States).

### Generation of *ap2* Quadruple Mutants of *B. napus* by CRISPR/Cas9-Mediated Genome Editing System

To construct the desired recombinant plasmids using CRISPR/Cas9-based system, the same target sequence of *AP2* for the four cDNAs of *B. napus* was designed using the web tool CRISPR-P^3^. The target dsDNA molecules with sticky ends were generated via direct annealing of two oligonucleotides primers in which the sticky ends sequences match those of the *Bsa* I ends in the pHSN401 vector (Xing et al., 2014). The resultant pHSN401-*AP2* constructs were transformed into the *Agrobacterium tumefaciens* strain GV3101:*pSoup* using electroporation, and subsequently transformed into *B. napus* by the previously described method (Bhalla and Singh, 2008). The genomic DNA extracted from T1 plants was used for PCR amplification with primer sets AP2-F250/AP2-R507, and the PCR products were cloned into pMD19-T Vector (Takara) for sequencing using M13-47 primer (Beijing Sunbiotech Co., Ltd.).

## Results

### Origin and Floral Identities of *scm*, a Novel Flower Mutant of *B. rapa*

**FIGURE 1:**
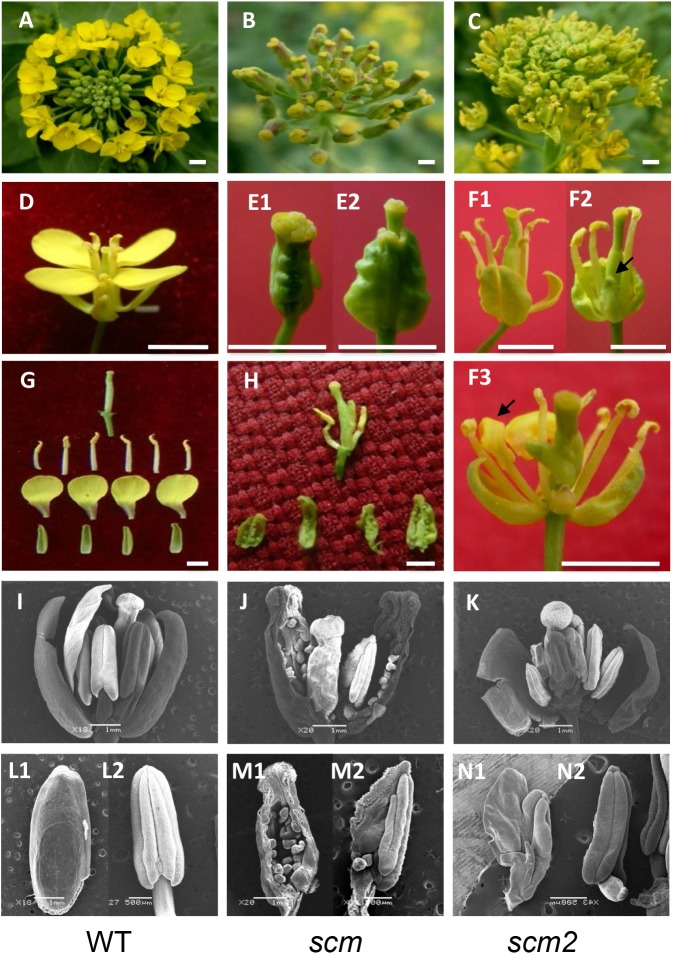
Characteristics the sepal carpeloid mutants of *Brassica.* **(A–C)**: inflorescence; **(D**,**E1**,**E2**,**F1–F3)**: flower; **(G,H)**: floral organs; **(I–K)**: SEM photos of flowers; **(L1,L2,M1,M2,N1,N2)**: SEM photos of floral organs; **(A,D,G,I,L1,L2)**: wild type *Brassica* (WT). **(L1**,**L2)** Are sepal (left) and stamen (right); **(B,E1,E2,H,J,M1,M2)**: the typical sepal carpeloid mutant (*scm*). **M1** is a sepal carpeloid organ, and **M2** is an incomplete stamen to carpel organ with one ovule; **(C,F1–F3,K,N1,N2)**: the incomplete sepal carpeloid mutant (*scm2*). Arrows indicate sepal carpel-like transition in **F2** and petal stamen-like transition in **F3**. **N1** is an incomplete petal to stamen organ, and **N2** is a stamen of *scm*2. Bars in **E1** and **E2** are 5 mm; bars in **I–K,L1,M1** are 1 mm; bars in **L2,M2,N1,N2** are 500 μm; and bars in the others are 10 mm.

During our breeding experiments, we recovered a novel pistil-like flower mutant in the F_3_ selfing line Nc116, which was generated from an interspecific cross between *B. napus* and *B. rapa*. Unlike the wild-type *Brassica* (**Figures 1A,D,G,I,L1,L2**, WT), the flowers of the mutant consisted of a very large pistil and lacked sepals, petals, or stamens (**Figures 1B,E1**). After 2 days of growth, the mutants showed a small pistil stretching out of the large, initial pistil (**Figure 1E2**). When the large pistil was torn, it appeared to be separated into three parts: the outer layer was a large pistil consisting of four fused carpels with green ovules inside; the intermediate layer only consisted of only 1–4 abnormal stamens with chimeric carpel-like homeotic identity; and the inner layer was a normal pistil (**Figures 1H,J,M1,M2**). Surprisingly, it appeared that the four whorls and four types floral organs (sepals, petals, stamens, and carpels) found in normal flowers had transformed into only three whorls containing just two types of organs (carpels, stamens, and carpels, respectively), with the petals being completely abolished. Clearly, the mutant had severe defects in both the sepal and petal whorls; the ring of four merged carpels developed at the four sepal positions representing a typical homeotic transition of sepals to carpels, and the complete loss of the four petals indicating a typical apetalous trait. Evidently, the mutant had typical sepal carpeloid and apetalous characteristics, which we therefore designated as *scm*. Due to the reduced number of stamens in the *scm* flower compared to wild-type *Brassica* flowers, we could not conclude whether *scm* had undergone a homeotic transition of petals to stamens.

We also obtained another incomplete sepal carpeloid mutant, named *scm2*, in the same population. Compared to *scm*, this mutant lost the large pistil shape (**Figures 1C,K**), and only a few sepals developed into carpels (**Figures 1F1,F2**). Moreover, some flowers of *scm2* were also apetalous, some of which containing few small abnormal petals, and some showing stamen-like petals (**Figures 1F3,N1,N2**). These results indicated that *scm2* was an incomplete mutant with the homeotic transitions of sepals to carpels and petals to stamens.

### Expression of ABCE Class of Genes in the *scm* Mutant

According to the classical ABC model, floral organ identity genes, especially those of class A and B, specify sepal and petal development. Mutations in the A class gene *AP2* in *Arabidopsis* lead to homeotic transitions of sepals to carpels and petals to stamens (Kunst et al., 1989; Coen and Meyerowitz, 1991). Consistent with the previous findings, our mutants displayed a similar homeotic transition. This prompted us to examine the *AP2* gene expression pattern, as well as the expression of the other ABCE classes of genes, in *scm* and in wild-type *B. rapa* (WTr) and *B. napus* (WTn).

**FIGURE 2:**
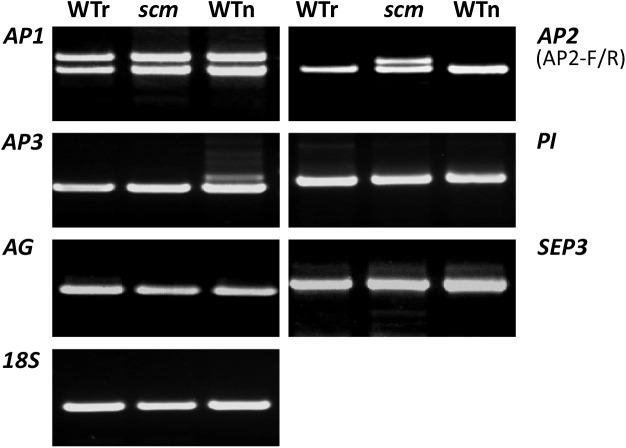
Expression of ABCE classic genes in the *scm* mutant and wild-type *Brassica.* WTr, wild-type *B. rapa*; WTn, wild-type *B. napus*; *scm*, sepal carpeloid mutant of *Brassica*.

Thus, the expression of *AP1*, *AP2*, *AP3*, *PI*, *AG*, and *SEP3* genes in these samples was studied by semi-quantitative RT-PCR. When *AP1*-specific primers were applied, all the three samples formed two identical PCR amplicons (**Figure 2**, top left). By contrast, when *BrAP2*-specific primers were used, both WTr and WTn each produced one clear band for amplicons of identical size, whereas *scm* formed two bands (**Figure 2**, top right). The length of one of the bands was similar to both controls, while the other band was slightly larger than its counterpart. This larger fragment was named *SCM-a*, and the smaller fragment was defined as *SCM-b*. Using *AP3* primers, we detected fragments of the same size in the WTr and *scm* samples, but an additional larger band appeared in the gel of the WTn sample (**Figure 2**, second from the top left). Meanwhile, the amplification with *PI*, *AG*, and *SEP3* primers resulted in the production of identical amplicons in all the three samples, with no significant differences (**Figure 2**). These data indicate that a *Brassica BrAP2* mutation, like the sepal carpeloid *AP2*-gene mutant in *Arabidopsis*, is probably relevant to the *scm* mutant. Moreover, the results from the *AP3* gene amplification indicated that the genetic background of *scm* was very similar to that of its *B. rapa* parent.

### The Two *AP2* Orthologs in *B. rapa* Are Mutated

To explore the *AP2* orthologs in *B. rapa*, a BLAST search was performed in the *Brassica* database, and two genes, *Bra011741* and *Bra017809*, were identified. *Bra011741* is located on chromosome A01 between base pairs 00793728 and 00795868, while *Bra017809* is found on the chromosome A03 between base pairs 30612490 and 30614651. We also detected two *AP2* orthologs in *B. oleracea*, *Bol028934* and *Bol018627*, which are located on chromosomes C01 and C06, respectively (Supplementary Figure 1). Our results showed that *B. rapa* has two *BrAP2* genes, whereas *B. napus* might have four *BnAP2* genes, two of which from *B. rapa* and two from *B. oleracea* (Parkin et al., 1995; Snowdon et al., 2002).

**FIGURE 3:**
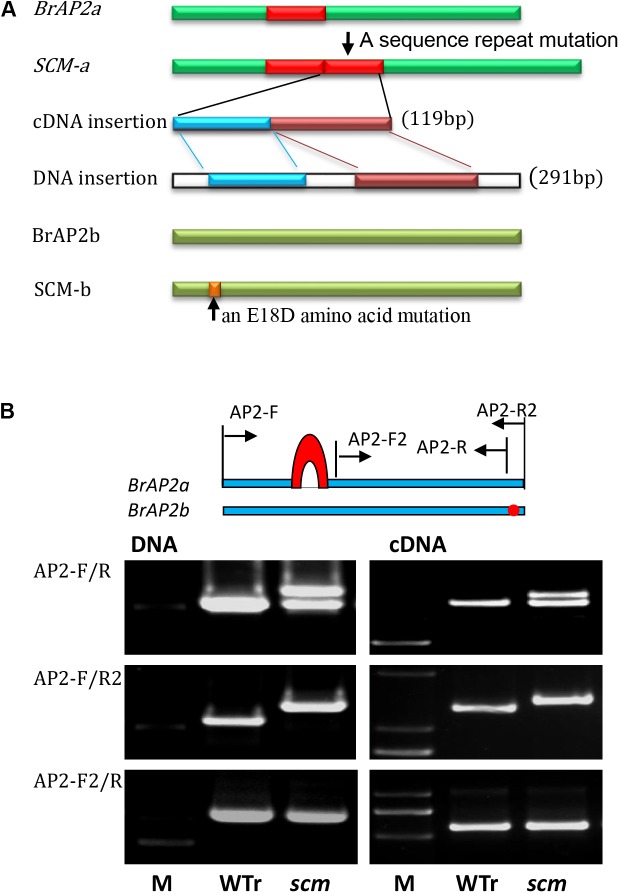
Mutations of the two *AP2* orthologs in the *scm* of *B. rapa.* **(A)** Schematic of the two *AP2* gene mutations in *scm*. The *BrAP2a* cDNA from *scm* (*SCM-a*) had a 119-bp sequence repeat mutation, which was derived from the transcription of a 291-bp repeat in genomic DNA that consisted of nucleotides 648–938 repeated after nucleotide 938. *BrAP2b* cDNA of *scm* (*SCM-b*) had an A to C single nucleotide mutation that transformed the 18th glutamic acid into aspartic acid (E18D). **(B)** A schematic of the location of the designed primers. The relative locations of the *AP2* primers in the *scm* and wild-type *B. rapa* (WTr) sequences are shown (upper part). PCR amplification of genome DNA and cDNA of WTr and the *scm* using primer pairs of AP2-F/R, AP2-F/R2, and AP2-F2/R (lower part).

According to the *BrAP2* sequences from *B. rapa* and *B. oleracea*, primers spanning the full length cDNA of the two *BrAP2* genes were designed: BrAP2-F and BrAP2-R2a target to the *Bra011741* and *Bol028394* genes; and BrAP2-F and BrAP2-R2b specific for the *Bra017809* and *Bol018627* genes. PCR products obtained from WTr samples using AP2-F/R2a primers were 1302-bp in length, and were named *BrAP2a*, sharing 99.4% nucleotide identity and 99.1% amino acid identity with *Bra011741*. Using the same AP2-F/R2a primer pair, the amplified genes from *scm* were 100% identical to *BrAP2a* except for a 119-bp insertion, which was a repeat of the sequence from nucleotides 485–603 (**Figure 3A** and Supplementary Figure 2).

The amplicons obtained from WTr samples using the BrAP2-F/R2b primer set were identified as a 1299-bp fragment named *BrAP2b.* This gene was 100% identical to *Bra017809*. The corresponding sequences from *scm* were 100% identical to *BrAP2b* except for two single-nucleotide substitutions, among which only one A to C mutation, translating into the alteration of the 18th glutamic acid of the corresponding protein into aspartic acid, was observed (**Figure 3A** and Supplementary Figure 3 E18D). Among all the tested *AP2* sequences obtained from *scm*, we could not detect any *AP2* genes derived from *B. oleracea*, thus confirming that the genetic background of *scm* was very similar to its *B. rapa* parent.

The amplification of genomic DNA using the AP2-F/R2a primer set revealed that the *BrAP2a* gene of *scm* had an additional 291-bp fragment, which was a repeat of *BrAP2a* nucleotides 648–938, inserted after nucleotide 938. The above-mentioned 119-bp cDNA insertion was derived from transcription of this 291-bp DNA repeat sequence (**Figure 3A** and Supplementary Figure 2). Further sequence analysis indicated that this insertion led to an early translation termination of the *BrAP2a* mRNA.

We then designed additional specific primers to further confirm the above results. Using the AP2-F/R primer set, both DNA and cDNA of *scm* displayed an additional band compared to WTr (**Figure 3B**). When the *BrAP2a* specific primer set AP2-F/R2a was applied, both DNA and cDNA of *scm* produced one band that was larger than that of WTr. Using a mutual primer set (AP2-F2/R) for both of the two *AP2* genes, both DNA and cDNA of *scm* formed one amplicon, the band for which was the same as that of the WTr-derived product. These results showed that the *BrAP2a* gene of *scm* underwent an insertion mutation that led to the loss of function of this gene. Altogether, these results suggested that mutations in *BrAP2a* and *BrAP2b* are most likely associated with the sepal carpeloid mutant *scm*. However, it was still unclear whether the loss of function of the two *AP2* genes was the key cause of the observed *scm* phenotype.

### The Two *AP2* Genes of *B. rapa* Can Both Rescue the *ap2-5* Mutant of *Arabidopsis*

The loss of function of *AP2* in *Arabidopsis* has been previously demonstrated to cause a homeotic transition of sepals to carpels and petals to stamens (Kunst et al., 1989). Moreover, a weak *AP2-*defective *Arabidopsis* mutant *ap2-5* shows a few sepal-to-carpel and petal-to-stamen homeotic transitions (Kunst et al., 1989). Both *AP2* genes in *B. rapa*, *BrAP2a* and *BrAP2b*, shared high similarity with the *Arabidopsis AP2* gene in their protein coding sequences (Supplementary Figure 3). To clarify whether *BrAP2a* and/or *BrAP2b* have biologically significant roles in floral organ specification, we transformed the two *B. rapa AP2* genes and *Arabidopsis AP2 (AtAP2*) gene (control) separately into the wild-type and *ap2-5* mutant *Arabidopsis*.

**FIGURE 4:**
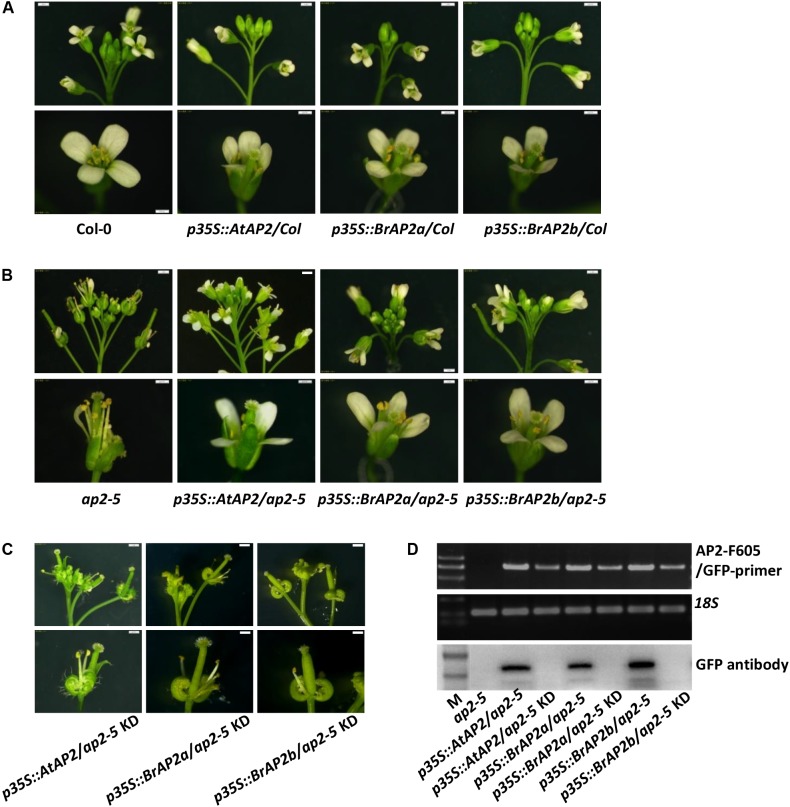
Wild type and an *ap2* weak mutant of *Arabidopsis* transformed with *AP2* genes from *B. rapa.* **(A)** Wild-type *Arabidopsis* (Col-0) transformed with *AtAP2* (control) and two *AP2* genes of *B. rapa*. Their over-expressed lines *p35S:AtAP2/Col*, *p35S::BrAP2a/Col*, and *p35S::BrAP2b/Col* had normal flowers similar to the wild-type flower. **(B)** The *AP2* mutant of *Arabidopsis* (*ap2-5*), which has a mild sepal-to-carpel and petal-to-stamen phenotype, transformed with *AtAP2* gene and two *B. rapa AP2* genes. All three transgenic plants, *p35S:AtAP2/ap2-5*, *p35S::BrAP2a/ap2-5*, and *p35S::BrAP2a/ap2-5*, rescued the *ap2-5* mutant defect and developed normal sepals and petals. **(C)** The knockdown lines, *p35S::AtAP2/ap2-5* KD, *p35S::BrAP2a/ap2-5* KD, and *p35S::BrAP2b/ap2-5* KD, from three transgenic group of *p35S:AtAP2/ap2-5*, *p35S::BrAP2a/ap2-5*, and *p35S::BrAP2a/ap2-5*. **(D)** semi-quantitative RT-PCR and Western blot assays to validate the transgenic lines. Bars, 500 μm.

All the three *AP2* genes were fused to green fluorescent protein (GFP) under the control of constitutive 35S promoter and transferred into *Arabidopsis* Col-0 to generate *p35S::AtAP2/Col*, *p35S::BrAP2a/Col*, and *p35S::BrAP2b/Col* over-expressing transgenic lines. These three transgenic lines displayed normal flower phenotypes (**Figure 4A**), suggesting that over-expression of the *AP2* gene does not affect floral organ development.

When the three *AP2* fusion constructs were transformed into the *ap2-5* mutant of *Arabidopsis*, the transgenic lines *p35S::AtAP2/ap2-5*, *p35S::BrAP2a/ap2-5*, and *p35S::BrAP2b/ap2-5* displayed normal development of the sepals and petals (**Figure 4B**), and thus the *AP2* defect was rescued. Moreover, we recovered some strong sepal carpeloid mutants not only in the three knockdown transgenic groups: *p35S::AtAP2/ap2-5* KD, *p35S::BrAP2a/ap2-5* KD, and *p35S::BrAP2b/ap2-5* KD (**Figure 4C**), but also in the three over-expressing groups caused by a cosuppression effect (data not shown). These mutants had some common characteristics: the sepals were transformed into carpels, the petals were absent, and the number of stamens was reduced to 1–4, but the pistil showed normal development. These lines displayed a strong *AP2*-knockdown phenotype, which is most likely also observed in *scm*. We further carried out RT-PCR and Western blot assays on these transgenic lines. Our RT-PCR results confirmed that the three *AP2* rescued lines exhibited high expression whereas the three knockdown phenotypes showed low expression level of the *AP2* gene. Interestingly, strong protein bands were present in the Western blot analyses of all the three *AP2* rescued lines but could not be detected for the three knock-down lines (**Figure 4D**). Therefore, we inferred that the exogenous *AP2* gene could interfere with the expression of endogenous *AtAP2* gene through RNAi effect. These results confirmed that the *AP2* gene is involved in the regulation of sepal and petal development, and the knockdown of these genes could affect sepal and petal development to generate sepal carpeloid and apetalous mutants.

### The *ap2* Quadruple Mutants of *B. napus* Produced by CRISPR/Cas9 System Resulting in Typical Sepal Carpeloid Phenotype

**FIGURE 5:**
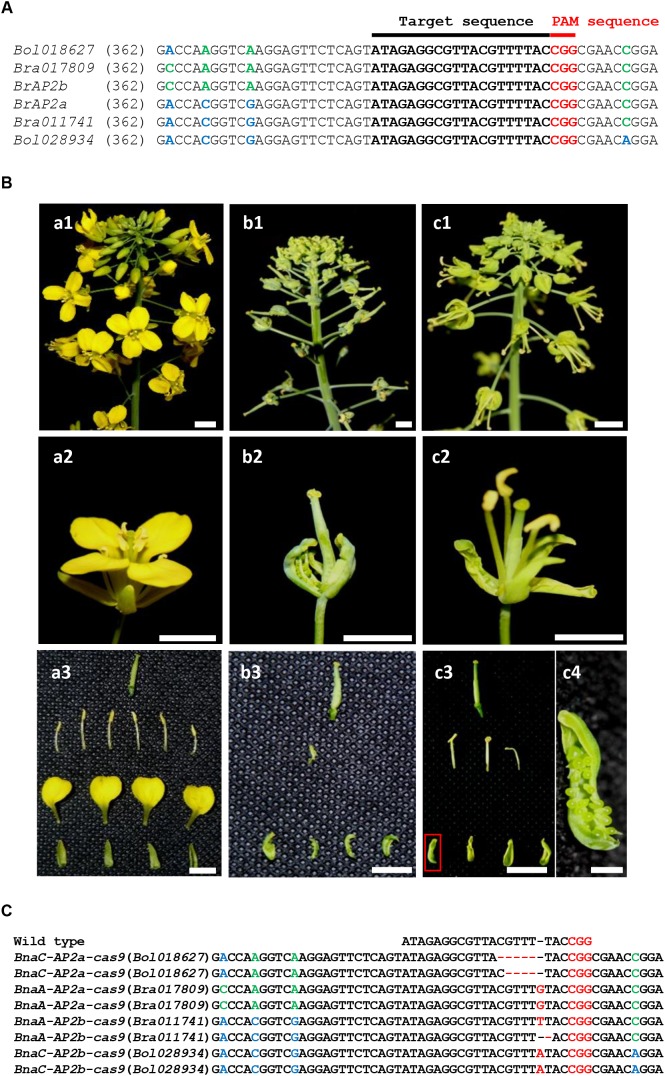
*ap2* quadruple mutant in *B. napus* generated by CRISPR/Cas9 genome editing system. **(A)** The designed target sequence used for CRISPR/Cas9. The PAM sequence is indicated in red. **(B)** a1–a3: wild-type *Brassica* (WT); b1–b3: a strong *ap2* quadruple mutant; c1–c4: a weak *ap2* quadruple mutant. a1, b1, c1: inflorescence; a2, b2, c2: flower; a3, b3, c3: floral organs; c4: enlargement of the floral organ shown in the red box in c3. The *ap2* quadruple mutant of *B. napus*, which has a similar sepal-to-carpel and petal-to-stamen phenotype as the *scm* mutant of *Brassica*. **(C)** Sequencing results of the target sites in the strong *ap2* mutant. Bars are 1 mm in c4 and 10 mm in all the others panels.

To further confirm that the *AP2* genes are involved in regulating sepal and petal development, a CRISPR/Cas9-mediated genome editing experiment was performed to test whether the loss of *AP2* function in *B. napus* could lead to a similar sepal carpeloid phenotype as in *scm*. We designed and constructed CRISPR/Cas9 nuclease system to cut at four identical target sites in the conserved sequences of *AP2* (**Figure 5A** and Supplementary Figure 4A).

After that, we performed *Agrobacterium*-mediated transformation to introduce CRISPR/Cas9 construct into *B. napus* to generate 33 transgenic plants (Yan et al., 2012). Among these, some plants showed typical sepal carpeloid phenotypes and some exhibited weak carpeloid phenotypes (**Figure 5B**). Furthermore, the genetic composition of one typical mutant was identified by sequencing, in which five or four nucleotides were deleted in each single chromosome of *BnaC-AP2a* gene; a “G” nucleotide was inserted into *BnaA-AP2a*; a “T” nucleotide was inserted or deleted in each single chromosome of *BnaA-AP2b* gene; and a “A” nucleotide was inserted into *BnaC-AP2b* (**Figure 5C** and Supplementary Figure 4B). In this way, all of the four *AP2* genes in *B. napus* acquired reading frameshift mutations and early translation termination, reflecting the fact that the four *AP2* genes completely lost their functions. The resulting *ap2* quadruple mutant exhibited carpels sepals, missing petals, and a reduced number of stamens, but showed a normal pistil, which are identical phenotypes to those displayed by *scm* (**Figure 1B**). These results suggest that knocking out the *AP2* genes could severely affect floral organ development and lead to the generation of a sepal carpeloid and apetalous mutant in *Brassica*.

## Discussion

### The Novel Pistil-Like Flower Mutant Is a Sepal Carpeloid and Apetalous Mutant

The *scm* displayed some distinct characteristics: the sepals and petals were absent; the four merged carpels that looked like a very large pistil developed in the sepal positions; stamens reduced in numbers to 1–4; and only the pistil developed normally. Clearly, the mutant underwent two substantial changes with respect to its floral organs: one was a typical homeotic transformation of sepals into carpels, and the other was an apetalous phenotype. Thus, the pistil-like flower mutant was not only a typical sepal carpeloid mutant but also an apetalous mutant.

Moreover, we found some petals undergoing transformation into stamens in *scm2* indicating that the *scm2* underwent an incomplete homeotic transition of sepals to carpels and petals to stamens. It has been well-documented in *Arabidopsis* that the defective A-function *AP2* gene affects sepal and petal development and leads to the homeotic transformation of sepals to carpels and petals to stamens (Kunst et al., 1989). However, our weak and strong mutants presented different phenotypes in which the weak mutant exhibited an incomplete transformation of sepals to carpels and petals to stamens, whereas the strong mutant exhibited a complete sepal carpeloid transition and an apetalous trait. These results suggest that the *AP2* strong mutant generates only the sepal-carpeloid transformation and the missing petals trait, but could not produce the petal-to-stamen transformation. Although the phenotypes of the two mutants were not identical, both had severe sepal and petal developmental defects.

### Defective *AP2* Genes Are the Main Cause of the *scm* Phenotype

Our data revealed that the *scm* mutant, derived from the F_3_ generation of an interspecific cross between *B. napus* and *B. rapa*, contains only two, *B. rapa-*similar *AP2* genes, suggesting that its genetic background is close to that of the parent *B. rapa*. Both the *AP2* genes in *scm* were mutated: one contained a partial repeat sequence that led to an early translation termination signal, and the other included an A to C single-nucleotide substitution that transformed the 18th glutamic acid of the corresponding protein into an aspartic acid. This mutation occurred in a highly conserved acidic serine-rich region (amino acids 14–50) that may serve as a transcriptional activation domain (Jofuku et al., 1994). Overall, the mutation of both *AP2* genes in *scm* most likely resulted in the loss of function of these genes. Correspondingly, Yan et al. (2012) showed that knocking out the *BnAP2* gene in *B. napus* generated a sepal carpeloid and apetalous mutant as well as some incomplete mutants. Similarly, we also generated several *AP2*-knockdown plants in the *AP2*-overexpressing transgenic *Arabidopsis* lines, as well as a number of *AP2*-knockout *B. napus* transgenic plants with a sepal carpeloid and apetalous phenotype. These results further confirmed that loss-of-function mutations of the *AP2* genes resulted in the sepal carpeloid and apetalous phenotypes.

Our transgenic results demonstrated that the two normal *AP2* genes from *B. rapa* can both rescue the sepal and petal developmental defects of the *ap2-5* mutant of *Arabidopsis*, indicating that both normal *AP2* genes have the ability to regulate sepal and carpel development, and reflecting that mutation of neither *BrAP2a* nor *BrAP2b* alone can lead to the *scm* phenotype.

Taken together, our results confirm that the *AP2* gene plays a key role in regulating sepal and petal development and that its function is highly conserved in Cruciferae family, many of which are important crop plants. The complete loss of AP2 function severely affects sepal and petal development and generates sepal carpeloid and apetalous mutants. The two defective *AP2* genes of *B. rapa* were the core cause for the sepal carpeloid mutant *scm* phenotype. However, the molecular mechanism of floral development regulated by *AP2* gene is still unclear, and needs to be investigated further.

## Author Contributions

XiW and YZ designed the experiments. YZ, SH, JL, JM, and XG performed the experiments. YZ and SH wrote and revised the article. XuW and JT bred the *scm* mutant. All the authors gave the final approval for submission of the manuscript.

## Conflict of Interest Statement

The authors declare that the research was conducted in the absence of any commercial or financial relationships that could be construed as a potential conflict of interest.

## Footnotes

1 www.arabidopsis.org

2 http://brassicadb.org/brad/

3 http://crispr.hzau.edu.cn/CRISPR/
