# Supplementary material for: Defective APETALA2 Genes Lead to Sepal Modification in Brassica Crops
Source: Front Plant Sci. 2018 Mar 20;9:367. doi: 10.3389/fpls.2018.00367 (PMC5869249; doi:10.3389/fpls.2018.00367)
Supplement: Supplementary file 1 [file Table_1.PDF]

Supplemental Table 1

Primers List

Purpose	Name	Primer sequence (5'-3')	Gene
RT-PCR	18S-F	ACGATGAGTGTTCGCCCTTGG	*18S*
	18S-R	TTCCTTTGAGTTTCGGTCTTGC	
	AP1- F65	TCGAAAAGAAGAGCTGGTCT	*AP1*
	AP1-R751	AGCCAAGGTTGCAGTTGTAAA	
	AP2F	ATGGGTCAATTCTTAGGC	*AP2*
	AP2 R	TCAAGAAGGTCTCATGAGAG	
	AP3-F	ATGGCGAGAGGGAAGATCCA	*AP3*
	AP3-R	TTCAAGAAGGTGGAAGGTAATGAT	
	PI-F	ATGGGAAGAGGGAAGATAGAGATA	*PI*
	PI-R	TCAATCGATGACCAAMGACAT	
	AG F	ATGGCTTACCAAATGGAG	*AG*
	AG R	TTACACTAACTGAAGAGCGGT	
	sep3 F	ATGGGAAGAGGGAGAGTAG	*SEP3*
	SEP3 R	TCAAATAGAGTTGGTGTCATAAGG	
AP2-special primers	AP2-F2	GGTTTGTTCGACACCGAGGT	*AP2*
	AP2-R2a	AGAAGGTCTCATGAGAGAA	
	AP2-R2b	AGGTCTCATGAGAGGAGG	
Cloning primers	BAP2-F+Sac I	ACGAGCTCATGTGGGATCTAAACGACTCACC	*AP2*
	BAP2-R2a+Xba I	ACTAGTCTAGAAGAAGGTCTCATGAGAGAAGGTTG	
	BAP2-R2b+Xba I	ACTAGTCTAGAAGGTCTCATGAGAGGAGGTTGG	
	AtAP2-F+KpnI	CGGGGTACCATGTGGGATCTAAACGACGC	
	AtAP2-R+XbaI	CTAGTCTAGAAGAAGGTCTCATGAGAGGAGG	
RT-PCR	BraAP2-F605	GACGAATTTAACGAAGGAAGAG	
	GFP-primer	ACCACCCCGGTGAACAGC	
	Actin2-F388	AACTCTCCCGCTATGTATGTCG	
	Actin2-R573	GAGGTAATCAGTAAGGTCACGTCC	
CRISPR/Cas9 to target AP2	AP2-sgRNA-F	ATTGATAGAGGCGTTACGTTTTAC	
	AP2-sgRNA-R	AAACGTAAAACGTAACGCCTCTAT	
AP2 genotyping	AP2-F250	GGCTTTCCTCGGTCTCACTG	
	AP2-R507	TGCATGAGCAGTGTCAAATCC	
