# Supplementary material for: Defective APETALA2 Genes Lead to Sepal Modification in Brassica Crops
Source: Front Plant Sci. 2018 Mar 20;9:367. doi: 10.3389/fpls.2018.00367 (PMC5869249; doi:10.3389/fpls.2018.00367)
Supplement: Supplementary file 2 [file Image_1.PDF]

Supplemental Figure 1

Nucleotide alignments of the two *AP2* genes in *Brassica rapa*

```
                 1                                                                                        90
Bol018627   (1)  ATGTGGGATCTAAACGACTCACCACACCAGACAGTCAGACAAGAAGAATCTGAAGAGTTTTGTTATTCTTCACCGGGTAAACGGGTCGGA
Bra017809   (1)  ATGTGGGATCTAAACGACTCACCACACCAAACAGTCAGACAAGAAGAATCTGAAGAGTTTTGTTATTCTTCACCGGGTAAACGGGTCGGA
BrAP2b      (1)  ATGTGGGATCTAAACGACTCACCACACCAAACAGTCAGACAAGAAGAATCTGAAGAGTTTTGTTATTCTTCACCGGGTAAACGGGTCGGA
BrAP2a      (1)  ATGTGGGATCTAAACGACTCACCACACCAAACACTTGTTGAAGAAGAATCTGAAGAGTTGTGTTATTCTTCACCGGGTAAACGGGTCGGA
Bra011741   (1)  ATGTGGGATCTAAACGACTCACCACACCAAACACTTGTTGAAGAAGAATCTGAAGAGTTGTGTTATTCTTCACCGGGTAAACGGGTCGGA
Bol028934   (1)  ATGTGGGATCTAAACGACTCACCACACCAAACACTTGTTGAAGAAGAATCTGAAGAGTTGTGTTATTCTTCACCGGGTAAACGGGTCGGA
                 91                                                                                      180
Bol018627  (91)  TCTTTCTCAAACTCAAGTTCATCTGCTGTAGTCATTGAAGATGGATCCGATGATGATGAACCTAACCGGGTTAGAGCCAACAACCCTCTT
Bra017809  (91)  TCTTTCTCAACCTCAAGTTCATCTGCTGTAGTCATCGAAGATGGATCCGATGACGATGAACCTAACCGGGTCAGACCCAACAACCCTCTT
BrAP2b     (91)  TCTTTCTCAACCTCAAGTTCATCTGCTGTAGTCATCGAAGATGGATCCGATGACGATGAACCTAACCGGGTCAGACCCAACAACCCTCTT
BrAP2a     (91)  TCTTTCTCGAACTCAAGTTCATCTGCTGTAGTCATCGAAGATGGATCCGATGATGATGAGCCTAACCGGGTCAGACCCAACAACCCACTT
Bra011741  (91)  TCTTTCTCGAACTCAAGTTCATCTGCTGTAGTCATCGAAGATGGATCCGATGATGATGAGCACAACCGGGTCAGACCCAACAACCAACTT
Bol028934  (91)  TCTTTCTCGAACTCGAGTTCATCTGCTGTAGTCATCGAAGATGGATCCGATGATGATGAGCCTAACCGGGTCAGACCCAACAACCCACTT
                 181                                                                                     270
Bol018627 (181)  ATAACCCATCAGTTCTTCCCGGAGATGGAATCTAGCGTCGGAGACAACGGTGGTGGTGGTCCCGGGTCGAGCTTTCCTCGGTCTCACTGG
Bra017809 (181)  ATCACCCATCAGTTCTTCCCGGAGATGGAATCTAGCGTCGGAGACAACGGTGGTGGTGGTACCGGGCCGGGCTTTCCTCGGTCTCACTGG
BrAP2b    (181)  ATCACCCATCAGTTCTTCCCGGAGATGGAATCTAGCGTCGGAGACAACGGTGGTGGTGGTACCGGGCCGGGCTTTCCTCGGTCTCACTGG
BrAP2a    (181)  GTCACCCATCAGTTCTTCCCACAGATGGAAACTAGCGTAGGGGATGATGGTGGAGGTGGTCCCGGGTCGGGCTTTCCTCGGTCTCACTGG
Bra011741 (181)  GTCACCCATCAGTTCTTCCCACAGATGGAAACGAGCGTAGGGGACGATGGTGGAGGTGGTCCCGGGTCGGGCTTTCCTCGGTCTCACTGG
Bol028934 (181)  GTCACCCATCAGTTCTTCCCAGAGATGGAAACTAGCGTAGGGGACGATGGTGGTGGTGGTCCCGGGTCGGGCTTTCCTCGGTCTCACTGG
                 271                                                                                     360
Bol018627 (271)  TTTGGTGTACAGTTCTCTCAGTCGGACCTAGCTACAGGATCATCGGTAGGTAAACCCGCAACTGTTGGTGCGGTGGTGGAGCCGGCACAG
Bra017809 (271)  TTTGGTGTACAGTTTTCTCAGTCGGATCTAGCTACCGGATCATCGGTAGGTAAACCCGCAACTGTTGGAGCGGTGGTGGAACCGACACAG
BrAP2b    (271)  TTTGGTGTACAGTTTTCTCAGTCGGATCTAGCTACCGGATCATCGGTAGGTAAACCCGCAACTGTTGGAGCGGTGGTGGAACCGACACAG
BrAP2a    (271)  TTTGGTGTTAAGTTTTGTCAGTCCGATCTAGCTACCGGATCATCAGCGGGTAAACCCGCCACTGTAGCCCCCGTGGCGGAGCCGGCTCAG
Bra011741 (271)  TTTGGTGTTAAGTTTTGTCAGTCCGATCTAGCTACCGGATCATCAGCGGGTAAACCCGCCACTGTAGCCCCCGTGGTGGAGCCGGCTCAG
Bol028934 (271)  TTTGGTGTAAAGTTTTGTCAGTCCGATCTAGCTACCGGATCATCGGCCGGTAAACCCGCCACTGTCGCCGCCGTGGTGGAGCCGGCTCAG
                 361                                                                                     450
Bol018627 (361)  CCGTTAAAAAAGAGCCGGCGTGGACCAAGGTCAAGGAGTTCTCAGTATAGAGGCGTTACGTTTTACCGGCGAACCGGAAGATGGGAGTCT
Bra017809 (361)  CCGTTAAAAAAGAGCCGGCGTGGCCCAAGGTCAAGGAGTTCTCAGTATAGAGGCGTTACGTTTTACCGGCGAACCGGAAGATGGGAGTCT
BrAP2b    (361)  CCGTTAAAAAAGAGCCGGCGTGGCCCAAGGTCAAGGAGTTCTCAGTATAGAGGCGTTACGTTTTACCGGCGAACCGGAAGATGGGAGTCT
BrAP2a    (361)  CCGTTGAAAAAGAGCAGGCGTGGACCACGGTCGAGGAGTTCTCAGTATAGAGGCGTTACGTTTTACCGGCGAACCGGAAGATGGGAATCT
Bra011741 (361)  CCGTTGAAAAAGAGCAGGCGTGGACCACGGTCGAGGAGTTCTCAGTATAGAGGCGTTACGTTTTACCGGCGAACCGGAAGATGGGAATCT
Bol028934 (361)  CCGTTGAAAAAGAGCAGGCGTGGACCACGGTCGAGGAGTTCTCAGTATAGAGGCGTTACGTTTTACCGGCGAACAGGAAGATGGGAATCT
                 451                                                                                     540
Bol018627 (451)  CATATTTGGGACTGTGGCAAACAGGTTTACTTAGGTGGATTTGACACTGCTCATGCAGCTGCTCGAGCATATGATAGAGCTGCTATTAAA
Bra017809 (451)  CATATTTGGGACTGTGGCAAACAGGTTTACTTAGGTGGATTTGACACTGCTCATGCAGCTGCTCGAGCATATGATAGAGCTGCTATTAAA
BrAP2b    (451)  CATATTTGGGACTGTGGCAAACAGGTTTACTTAGGTGGATTTGACACTGCTCATGCAGCTGCTCGAGCATATGATAGAGCTGCTATTAAA
BrAP2a    (451)  CATATTTGGGACTGTGGGAAGCAGGTTTACTTAGGTGGATTTGACACTGCTCATGCAGCTGCTCGAGCATATGATCGAGCTGCTATTAAA
Bra011741 (451)  CATATTTGGGACTGTGGGAAGCAGGTTTACTTAGGTGGATTTGACACTGCTCATGCAGCTGCTCGAGCATATGATCGAGCTGCTATTAAA
Bol028934 (451)  CATATTTGGGACTGTGGGAAGCAGGTTTACTTAGGTGGATTTGACACTGCTCATGCAGCTGCTCGAGCATATGATCGAGCTGCTATTAAA
                 541                                                                                     630
Bol018627 (541)  TTCCGTGGGGTAGAAGCTGATATCAATTTTAACATCGAAGATTATGATAATGACATGAAGCAGATGACGAATTTAACGAAGGAAGAGTTC
Bra017809 (541)  TTCCGTGGGGTAGAAGCTGATATCAATTTTAACATCGAAGATTATGATAATGACATGAAGCAGATGACGAATTTAACGAAGGAAGAGTTC
BrAP2b    (541)  TTCCGTGGGGTAGAAGCTGATATCAATTTTAACATCGAAGATTATGATAATGACATGAAGCAGATGACGAATTTAACGAAGGAAGAGTTC
BrAP2a    (541)  TTTCGTGGAGTAGAAGCTGATATCAACTTTACCATTGAAGATTATGATGATGACTTGAAGCAGATGACGAATTTAACGAAGGAAGAGTTC
Bra011741 (541)  TTTCGTGGAGTAGAAGCTGATATCAACTTTACCATTGAAGATTATGATGATGACTTGAAGCAGATGACGAATTTAACGAAGGAAGAGTTC
Bol028934 (541)  TTTCGTGGAGTAGAAGCTGATATCAACTTTACCATTGAAGATTATGATGATGACTTGAAGCAGATGACGAATTTAACGAAGGAAGAGTTC
                 631                                                                                     720
Bol018627 (631)  GTGCACGTACTTCGCCGACAAAGCACAGGGTTCCCTCGAGGAAGTTCGAAGTATAGAGGTGTCACTTTGCATAAGTGTGGTCGTTGGGAA
Bra017809 (631)  GTGCACGTACTTCGCCGACAAAGCACAGGGTTCCCTCGAGGAAGTTCGAAGTATAGAGGTGTCACTTTGCATAAGTGTGGTCGCTGGGAA
BrAP2b    (631)  GTGCACGTACTTCGCCGACAAAGCACAGGGTTCCCTCGAGGAAGTTCGAAGTATAGAGGTGTCACTTTGCATAAGTGTGGTCGCTGGGAA
BrAP2a    (631)  GTGCACGTACTTCGCCGACAAAGCACAGGCTTCCCTCGAGGCAGTTCAAAGTATAGAGGTGTCACTTTGCATAAGTGTGGTCGTTGGGAA
Bra011741 (631)  GTGCACGTACTTCGCCGACAAAGCACAGGCTTCCCTCGAGGCAGTTCAAAGTATAGAGGTGTCACTTTGCATAAGTGTGGTCGTTGGGAA
Bol028934 (631)  GTGCACGTACTTCGCCGACAAAGCACAGGCTTCCCTCGAGGCAGTTCAAAGTATAGAGGTGTTACTTTGCATAAGTGTGGTCGTTGGGAA
                 721                                                                                     810
Bol018627 (721)  GCTCGAATGGGCCAATTCTTAGGCAAAAAGTATGTTTATTTGGGTTTGTTCGACACCGAGGTTGAGGCTGCTAGAGCTTATGATAAAGCT
Bra017809 (721)  GCTCGAATGGGCCAATTCTTAGGCAAAAAGTATGTTTATTTGGGTTTGTTCGACACCGAGGTTGAGGCTGCTAGAGCTTATGATAAAGCT
BrAP2b    (721)  GCTCGAATGGGCCAATTCTTAGGCAAAAAGTATGTTTATTTGGGTTTGTTCGACACCGAGGTTGAGGCTGCTAGAGCTTATGATAAAGCT
BrAP2a    (721)  GCTCGAATGGGTCAATTCTTAGGCAAAAAGTATGTTTATTTGGGTTTGTTCGACACCGAGGTTGAAGCTGCTAGAGCTTACGATAAAGCT
Bra011741 (721)  GCTCGAATGGGTCAATTCTTAGGCAAAAAGTATGTTTATTTGGGTTTGTTCGACACCGAGGTTGAAGCTGCTAGAGCTTACGATAAAGCT
Bol028934 (721)  GCTCGAATGGGTCAATTCTTAGGCAAAAAGTATGTTTATTTGGGTTTGTTCGACACCGAGGTTGAAGCTGCTAGAGCTTACGATAAAGCT
                 811                                                                                     900
Bol018627 (811)  GCAATCAAATGTAATGGCAAAGATGCTGTGACTAACTTTGATTCAAGCATATACGACGAAGAACTGAATGCCGAGTCATCAGGGAATCCT
Bra017809 (811)  GCAATCAAATGTAATGGCAAAGATGCTGTGACTAACTTTGATTCAAGCATATACGACGAAGAACTGAATGCCGAGTCATCAGGGAATCCT
BrAP2b    (811)  GCAATCAAATGTAATGGCAAAGATGCTGTGACTAACTTTGATTCAAGCATATACGACGAAGAACTGAATGCCGAGTCATCAGGGAATCCT
BrAP2a    (811)  GCAATCAAATGTAATGGCAAAGACGCTGTGACTAACTTTGATCCAAGCATATACGACGACGAATTGAATGCCGAGTCATCAGGGAATCCT
Bra011741 (811)  GCAATCAAATGTAATGGCAAAGACGCTGTGACTAACTTTGATCCAAGCATATACGACGACGAATTGAATGCCGAGTCATCAGGGAATCCT
Bol028934 (811)  GCAATAAAATGTAATGGCAAAGACGCTGTGACTAACTTTGATCCAAGCATATACGACGACGAATTGAATGCTGAGTCATCAGGGAATTCT
```

```
                   901                                                                                      990
Bol018627   (901)  ATTCAACATGATCATAACCTCGATTTGAGTTTGGGAAACTCGGTTAATTCGAAGCATAATGGTCAAGATATGCGGCTCAAGATGAACCAA
Bra017809   (901)  ATTCAACAGGATCATAACCTCGATTTGAGTTTGGGAAACTCGGTTAATTCGAAGCATAAGGGTCAAGATATGCGGCTCAAGATGAACCAA
BrAP2b      (901)  ATTCAACAGGATCATAACCTCGATTTGAGTTTGGGAAACTCGGTTAATTCGAAGCATAAGGGTCAAGATATGCGGCTCAAGATGAACCAA
BrAP2a      (901)  ATTCAACAAGATCATAACCTCGATTTGAGTTTGGGAAACTCGGTTAATTCGAAGCAAAAGGGTCAAGATATGCGGCTCAAGATGAACCAA
Bra011741   (901)  ATTCAACAAGATCATAACCTCGATTTGAGTTTGGGAAACTCGGTTAATTCGAAGCAAAAGGGTCAAGATATGCGGCTCAAGATGAACCAA
Bol028934   (901)  ATTCAACAAGATCATAACCTCGATTTGAGTTTGGGAAACTCGGTTAATTCGAAGCAAAAGGGTCAAGATATGCGGCTCAAGATGAACCAA
                   991                                                                                     1080
Bol018627   (991)  CAAGATTCCCTCCACTCTAATGAGATTCTCGGTTTAGGTC---CCGGAATGGTTAACTATATCCCCGATTCTGTTCACCAATTTCCGGGC
Bra017809   (991)  CAAGATTCCCTCCACTCTAATGAGATTCTCGGATTAGGTCAAACCGGAATGGTTAACTATATCCCCAATTCTGTTCACCAATTTCCGGGC
BrAP2b      (991)  CAAGATTCCCTCCACTCTAATGAGATTCTCGGATTAGGTCAAACCGGAATGGTTAACTATATCCCCAATTCTGTTCACCAATTTCCGGGC
BrAP2a      (991)  CAAGATTCTCTTCATCCTAATGAGATTCTTGGATTGGGTCAAACCGGAATGGTTAACCATATCCCAAATTCAAATCTCCAATTTCGGGGC
Bra011741   (991)  CAAGATTCTCTTCATCCTAATGAGATTCTTGGATTAGGTCAAACCGGAATGGTTAACCATATCCCAAATTCAAATCTCCAATTTCCGGGC
Bol028934   (991)  CAAGAGACTCTTCATCCTAATGAGATTCTTGGATTAGGTCAAACCGGAATGGTTAACCATATCCCAAATTCAAATCTCCAATTTCCGGGC
                   1081                                                                                    1170
Bol018627  (1078)  AGCAGTAACATTCATGGCGGAGGAGGATTCTCGCTGTTTCCGGTGACCGAGAACCACCAGTTTGAAGGTCGGACCACCGCGAACCAAGTG
Bra017809  (1081)  AGCAGTAACATTCACCGCGGAGGAGGATTCTCGCTGTTTCCGGTGACCGAGAACCACCAGTTTGAAGGTCGGACCACCGCGAACCAAGTG
BrAP2b     (1081)  AGCAGTAACATTCACCGCGGAGGAGGATTCTCGCTGTTTCCGGTGACCGAGAACCACCAGTTTGAAGGTCGGACCACCGCGAACCAAGTG
BrAP2a     (1081)  AGCAGCAACATTGGTGGCGGAGGAGGATTCTCACTATTTCCGGTGGCTGAGAACCACCGGTTTGATGGTCGGACCACGACGAACCAAGTG
Bra011741  (1081)  AGCAGCAACATTGGTGGCGGAGGAGGATTCTCACTATTTCCGGTGGCTGAGAACCACCGGTTTGATGGTCGGACCACGACGAACCAAGTG
Bol028934  (1081)  AGCAGCAACATTGGTGGCGGAGGAGGATTCTCGCTATTTCCGGTGGCTGAGAACCACCGGTTTGATGGTCGGACCACGACGAACCAAGTG
                   1171                                                                                    1260
Bol018627  (1168)  TTGGCAAATGCTGCAGCATCATCAGGATTCTCTCCTCATAATCACCATCAGATTTTTAATTCTACTCCTACTTCTCATCAGAATTGGCTG
Bra017809  (1171)  TTGGCAAATGCTGCAGCATCATCAGGATTCTCTCCTCATAATCACAATCAGATTTTTAATTCTACTCCTACTTCTCATCAGAATTGGCTG
BrAP2b     (1171)  TTGGCAAATGCTGCAGCATCATCAGGATTCTCTCCTCATAATCACAATCAGATTTTTAATTCTACTCCTACTTCTCATCAGAATTGGCTG
BrAP2a     (1171)  TTGGCAAATGCTGCAGCATCATCAGGATTCTCTCCTCATCATCACAATCAGATTTTTAATTCCACTTCTACTTCTCATCAAAATTGGCTG
Bra011741  (1171)  TTGGCAAATGCTGCAGCATCATCAGGATTCTCTCCTCATCATCACAATCAGATTTTTAATTCCACTTCTACTTCTCATCAAAATTGGCTG
Bol028934  (1171)  TTGGCAAATGCTGCAGCATCATCAGGATTCTCTCCTCATCATCACAATCAGATTTTTAATTCCACTTCTACTTCTCATCAAAATTGGCTG
                   1261                                    1302
Bol018627  (1258)  CAGACCAATGGCTTCCAACCTCCTCTCATGAGACCT---TGA
Bra017809  (1261)  CAGACCAATGGCTTCCAACCTCCTCTCATGAGACCT---TGA
BrAP2b     (1261)  CAGACCAATGGCTTCCAACCTCCTCTCATGAGACCT---TGA
BrAP2a     (1261)  CAGACCAATGGCTTCCAACCTTCTCTCATGAGACCTTCTTGA
Bra011741  (1261)  CAGACCAATGGCTTCCAACCTTCTCTCATGAGACCTTCTTGA
Bol028934  (1261)  CAGACCAATGGTTTCCAACCTTCT------------------
```

Note: *Bra017809* and *Bra011741* are two *AP2* genes of *B. rapa* from *Brassica rapa* database. *Bol018627* and *Bol028934* are *AP2* genes of *B. oleracea* from *B. rapa* database. *BrAP2a* and *BrAP2b* are *AP2* genes of *B. rapa* derived from our PCR amplification.
