# Supplementary material for: Defective APETALA2 Genes Lead to Sepal Modification in Brassica Crops
Source: Front Plant Sci. 2018 Mar 20;9:367. doi: 10.3389/fpls.2018.00367 (PMC5869249; doi:10.3389/fpls.2018.00367)
Supplement: Supplementary file 3 [file Image_2.PDF]

Supplemental Figure 2

BrAP2a genes between the SCM mutant and wild type *B.rapa*

```
                       1                                                                              80
Bra011741 cDNA   (1)   ATGTGGGATCTAAACGACTCACCACACCAAACACTTGTTGAAGAAGAATCTGAAGAGTTGTGTTATTCTTCACCGGGTAA
Bra011741 DNA    (1)   ATGTGGGATCTAAACGACTCACCACACCAAACACTTGTTGAAGAAGAATCTGAAGAGTTGTGTTATTCTTCACCGGGTAA
BrAP2a cDNA      (1)   ATGTGGGATCTAAACGACTCACCACACCAAACACTTGTTGAAGAAGAATCTGAAGAGTTGTGTTATTCTTCACCGGGTAA
BrAP2a DNA       (1)   ATGTGGGATCTAAACGACTCACCACACCAAACACTTGTTGAAGAAGAATCTGAAGAGTTGTGTTATTCTTCACCGGGTAA
SCM-a cDNA       (1)   ATGTGGGATCTAAACGACTCACCACACCAAACACTTGTTGAAGAAGAATCTGAAGAGTTGTGTTATTCTTCACCGGGTAA
SCM-a DNA        (1)   ATGTGGGATCTAAACGACTCACCACACCAAACACTTGTTGAAGAAGAATCTGAAGAGTTGTGTTATTCTTCACCGGGTAA
                       81                                                                            160
Bra011741 cDNA   (81)  ACGGGTCGGATCTTTCTCGAACTCAAGTTCATCTGCTGTAGTCATCGAAGATGGATCCGATGATGATGAGCACAACCGGG
Bra011741 DNA    (81)  ACGGGTCGGATCTTTCTCGAACTCAAGTTCATCTGCTGTAGTCATCGAAGATGGATCCGATGATGATGAGCACAACCGGG
BrAP2a cDNA      (81)  ACGGGTCGGATCTTTCTCGAACTCAAGTTCATCTGCTGTAGTCATCGAAGATGGATCCGATGATGATGAGCCTAACCGGG
BrAP2a DNA       (81)  ACGGGTCGGATCTTTCTCGAACTCAAGTTCATCTGCTGTAGTCATCGAAGATGGATCCGATGATGATGAGCCTAACCGGG
SCM-a cDNA       (81)  ACGGGTCGGATCTTTCTCGAACTCAAGTTCATCTGCTGTAGTCATCGAAGATGGATCCGATGATGATGAGCCTAACCGGG
SCM-a DNA        (81)  ACGGGTCGGATCTTTCTCGAACTCAAGTTCATCTGCTGTAGTCATCGAAGATGGATCCGATGATGATGAGCCTAACCGGG
                       161                                                                           240
Bra011741 cDNA   (161) TCAGACCCAACAACCAACTTGTCACCCATCAGTTCTTCCCACAGATGGAAACGAGCGTAGGGGACGATGGTGGAGGTGGT
Bra011741 DNA    (161) TCAGACCCAACAACCAACTTGTCACCCATCAGTTCTTCCCACAGATGGAAACGAGCGTAGGGGACGATGGTGGAGGTGGT
BrAP2a cDNA      (161) TCAGACCCAACAACCCACTTGTCACCCATCAGTTCTTCCCACAGATGGAAACTAGCGTAGGGGATGATGGTGGAGGTGGT
BrAP2a DNA       (161) TCAGACCCAACAACCCACTTGTCACCCATCAGTTCTTCCCACAGATGGAAACTAGCGTAGGGGATGATGGTGGAGGTGGT
SCM-a cDNA       (161) TCAGACCCAACAACCCACTTGTCACCCATCAGTTCTTCCCACAGATGGAAACTAGCGTAGGGGATGATGGTGGAGGTGGT
SCM-a DNA        (161) TCAGACCCAACAACCCACTTGTCACCCATCAGTTCTTCCCACAGATGGAAACTAGCGTAGGGGATGATGGTGGAGGTGGT
                       241                                                                           320
Bra011741 cDNA   (241) CCCGGGTCGGGCTTTCCTCGGTCTCACTGGTTTGGTGTTAAGTTTTGTCAGTCCGATCTAGCTACCGGATCATCAGCGGG
Bra011741 DNA    (241) CCCGGGTCGGGCTTTCCTCGGTCTCACTGGTTTGGTGTTAAGTTTTGTCAGTCCGATCTAGCTACCGGATCATCAGCGGG
BrAP2a cDNA      (241) CCCGGGTCGGGCTTTCCTCGGTCTCACTGGTTTGGTGTTAAGTTTTGTCAGTCCGATCTAGCTACCGGATCATCAGCGGG
BrAP2a DNA       (241) CCCGGGTCGGGCTTTCCTCGGTCTCACTGGTTTGGTGTTAAGTTTTGTCAGTCCGATCTAGCTACCGGATCATCAGCGGG
SCM-a cDNA       (241) CCCGGGTCGGGCTTTCCTCGGTCTCACTGGTTTGGTGTTAAGTTTTGTCAGTCCGATCTAGCTACCGGATCATCAGCGGG
SCM-a DNA        (241) CCCGGGTCGGGCTTTCCTCGGTCTCACTGGTTTGGTGTTAAGTTTTGTCAGTCCGATCTAGCTACCGGATCATCAGCGGG
                       321                                                                           400
Bra011741 cDNA   (321) TAAACCCGCCACTGTAGCCCCCGTGGTGGAGCCGGCTCAGCCGTTGAAAAAGAGCAGGCGTGGACCACGGTCGAGGAGTT
Bra011741 DNA    (321) TAAACCCGCCACTGTAGCCCCCGTGGTGGAGCCGGCTCAGCCGTTGAAAAAGAGCAGGCGTGGACCACGGTCGAGGAGTT
BrAP2a cDNA      (321) TAAACCCGCCACTGTAGCCCCCGTGGCGGAGCCGGCTCAGCCGTTGAAAAAGAGCAGGCGTGGACCACGGTCGAGGAGTT
BrAP2a DNA       (321) TAAACCCGCCACTGTAGCCCCCGTGGCGGAGCCGGCTCAGCCGTTGAAAAAGAGCAGGCGTGGACCACGGTCGAGGAGTT
SCM-a cDNA       (321) TAAACCCGCCACTGTAGCCCCCGTGGCGGAGCCGGCTCAGCCGTTGAAAAAGAGCAGGCGTGGACCACGGTCGAGGAGTT
SCM-a DNA        (321) TAAACCCGCCACTGTAGCCCCCGTGGCGGAGCCGGCTCAGCCGTTGAAAAAGAGCAGGCGTGGACCACGGTCGAGGAGTT
                       401                                                                           480
Bra011741 cDNA   (401) CTCAGTATAGAGGCGTTACGTTTTACCGGCGAACCGGAAGATGGGAATCTCATATTTGG---------------------
Bra011741 DNA    (401) CTCAGTATAGAGGCGTTACGTTTTACCGGCGAACCGGAAGATGGGAATCTCATATTTGGTAATTTATTTGCAGTGAAAAA
BrAP2a cDNA      (401) CTCAGTATAGAGGCGTTACGTTTTACCGGCGAACCGGAAGATGGGAATCTCATATTTGG---------------------
BrAP2a DNA       (401) CTCAGTATAGAGGCGTTACGTTTTACCGGCGAACCGGAAGATGGGAATCTCATATTTGGTAATTTATTTGCAGTGAAAAA
SCM-a cDNA       (401) CTCAGTATAGAGGCGTTACGTTTTACCGGCGAACCGGAAGATGGGAATCTCATATTTGG---------------------
SCM-a DNA        (401) CTCAGTATAGAGGCGTTACGTTTTACCGGCGAACCGGAAGATGGGAATCTCATATTTGGTAATTTATTTGCAGTGAAAAA
                       481                                                                           560
Bra011741 cDNA   (460) ---------------------------------------------------------------------------GACTG
Bra011741 DNA    (481) ATATTCTTTAAATTTGATTAATCGATCGTTAATTAAAAGATAATGAATGTAAGATATGTTTCTCTATAATTCAGGGACTG
BrAP2a cDNA      (460) ---------------------------------------------------------------------------GACTG
BrAP2a DNA       (481) ATATTCTTTAAATTTGATTAATCGATCGTTAATTAAAAGATAATGAATGTAAGATATGTTTCTCTATAATTCAGGGACTG
SCM-a cDNA       (460) ---------------------------------------------------------------------------GACTG
SCM-a DNA        (481) ATATTCTTTAAATTTGATTAATCGATCGTTAATTAAAAGATAATGAATGTAAGATATGTTTCTCTATAATTCAGGGACTG
                       561                                                                           640
Bra011741 cDNA   (465) TGGGAAGCAGGTTTACTTAGGT----------------------------------------------------------
Bra011741 DNA    (561) TGGGAAGCAGGTTTACTTAGGTATATTAACTTTTTTTTGTTTTAGTTTTTTTTTTCATTTTTTTCTTGTATTCTGCTGTT
BrAP2a cDNA      (465) TGGGAAGCAGGTTTACTTAGGT----------------------------------------------------------
BrAP2a DNA       (561) TGGGAAGCAGGTTTACTTAGGTATATTAACTTTTTTTTGTTTTAGTTTTTTTTTTCATTTTTTTCTTGTATTCTGCTGTT
SCM-a cDNA       (465) TGGGAAGCAGGTTTACTTAGGT----------------------------------------------------------
SCM-a DNA        (561) TGGGAAGCAGGTTTACTTAGGTATATTAACTTTTTTTTGTTTTAGTTTTTTTTTTCATTTTTTTCTTGTATTCTGCTGTT
                       641                                                                           720
Bra011741 cDNA   (487) ------------------------------------------------------GGATTTGACACTGCTCATGCAGCTGC
Bra011741 DNA    (641) GAAAAATGTCATCATATAATTAAAAATATTTTTACCTTTTTTCCCTGAATAGGTGGATTTGACACTGCTCATGCAGCTGC
BrAP2a cDNA      (487) ------------------------------------------------------GGATTTGACACTGCTCATGCAGCTGC
BrAP2a DNA       (641) GAAAAATGTCATCATATAATTAAAAATATTTTTACCTTTTTTCCCTGAATAGGTGGATTTGACACTGCTCATGCAGCTGC
SCM-a cDNA       (487) ------------------------------------------------------GGATTTGACACTGCTCATGCAGCTGC
SCM-a DNA        (641) GAAAAATGTCATCATATAATTAAAAATATTTTTACCTTTTTTCCCTGAATAGGTGGATTTGACACTGCTCATGCAGCTGC
                       721                                                                           800
Bra011741 cDNA   (513) TCG-----------------------------------------------------------------------------
Bra011741 DNA    (721) TCGGTATGTTTCTGTCTTTGACTTGCTCTTTAACTCTTTTAATATCAAAAAACAAAAGGCACAAGACCTATAATATAAAG
BrAP2a cDNA      (513) TCG-----------------------------------------------------------------------------
BrAP2a DNA       (721) TCGGTATGTTTCTGTCTTTGACTTGCTCTTTAACTCTTTTAATATCAAAAAACAAAAGGCACAAGACCTATAATATAAAG
SCM-a cDNA       (513) TCG-----------------------------------------------------------------------------
SCM-a DNA        (721) TCGGTATGTTTCTGTCTTTGACTTGCTCTTTAACTCTTTTAATATCAAAAAACAAAAGGCACAAGACCTATAATATAAAG
```

Sequence	Start	801 … 880
Bra011741 cDNA	(516)	----------------------AGCATATGATCGAGCTGCTATTAAATTTCGTGGAGTAGAAGCTGATATCAACTTTACC
Bra011741 DNA	(801)	TCTTTTATTTTCTTTTATGTAGAGCATATGATCGAGCTGCTATTAAATTTCGTGGAGTAGAAGCTGATATCAACTTTACC
BrAP2a cDNA	(516)	----------------------AGCATATGATCGAGCTGCTATTAAATTTCGTGGAGTAGAAGCTGATATCAACTTTACC
BrAP2a DNA	(801)	TCTTTTATTTTCTTTTATGTAGAGCATATGATCGAGCTGCTATTAAATTTCGTGGAGTAGAAGCTGATATCAACTTTACC
SCM-a cDNA	(516)	----------------------AGCATATGATCGAGCTGCTATTAAATTTCGTGGAGTAGAAGCTGATATCAACTTTACC
SCM-a DNA	(801)	TCTTTTATTTTCTTTTATGTAGAGCATATGATCGAGCTGCTATTAAATTTCGTGGAGTAGAAGCTGATATCAACTTTACC
		881 … 960
Bra011741 cDNA	(574)	ATTGAAGATTATGATGATGACTTGAAGCAG--
Bra011741 DNA	(881)	ATTGAAGATTATGATGATGACTTGAAGCAG--
BrAP2a cDNA	(574)	ATTGAAGATTATGATGATGACTTGAAGCAG--
BrAP2a DNA	(881)	ATTGAAGATTATGATGATGACTTGAAGCAG--
SCM-a cDNA	(574)	ATTGAAGATTATGATGATGACTTGAAGCAG--
SCM-a DNA	(881)	ATTGAAGATTATGATGATGACTTGAAGCAGGTAACTCCTTAAACAAAACAAATAAAATGTCATCATATAATTAAAAATAT
		961 … 1040
Bra011741 cDNA	(604)	--
Bra011741 DNA	(911)	--
BrAP2a cDNA	(604)	--
BrAP2a DNA	(911)	--
SCM-a cDNA	(604)	-----------------------GTGGATTTGACACTGCTCATGCAGCTGCTCG--------------------------
SCM-a DNA	(961)	TTTTACCTTTTTTCCCTGAATAGGTGGATTTGACACTGCTCATGCAGCTGCTCGGTATGTTTCTGTCTTTGACTTGCTCT
		1041 … 1120
Bra011741 cDNA	(604)	--
Bra011741 DNA	(911)	--
BrAP2a cDNA	(604)	--
BrAP2a DNA	(911)	--
SCM-a cDNA	(635)	---AGCATAT
SCM-a DNA	(1041)	TTAACCCTTTTAATATCAAAAAACAAAAGGCACAAGACCTATAATATAAAGTCTTTTATTTTCTTTTATGTAGAGCATAT
		1121 … 1200
Bra011741 cDNA	(604)	--
Bra011741 DNA	(911)	--
BrAP2a cDNA	(604)	--
BrAP2a DNA	(911)	--
SCM-a cDNA	(642)	GATCGAGCTGCTATTAAATTTCGTGGAGTAGAAGCTGATATCAACTTTACCATTGAAGATTATGATGATGACTTGAAGCA
SCM-a DNA	(1121)	GATCGAGCTGCTATTAAATTTCGTGGAGTAGAAGCTGATATCAACTTTACCATTGAAGATTATGATGATGACTTGAAGCA
		1201 … 1280
Bra011741 cDNA	(604)	---A
Bra011741 DNA	(911)	-GTAACTCCTAAAACAAAACAAATAAAATACTAAACAATATGAATAGGTTATTATATTGATTAAAACGTAATTTAGCAGA
BrAP2a cDNA	(604)	---A
BrAP2a DNA	(911)	-GTAACTCCTTAAACAAAACAAATAAAATACTAATCAATATGAATAGGTTATTATATTGATTAAAACGTAATTTAGCAGA
SCM-a cDNA	(722)	G--A
SCM-a DNA	(1201)	GGTAACTCCTTAAACAAAACAAATAAAATACTAATCAATATGAATAGGTTATTATATTGATTAAAACGTAATTTAGCAGA
		1281 … 1360
Bra011741 cDNA	(605)	TGACGAATTTAACGAAGGAAGAGTTCGTGCACGTACTTCGCCGACAAAGCACAGGCTTCCCTCGAGGCAGTTCAAAGTAT
Bra011741 DNA	(990)	TGACGAATTTAACGAAGGAAGAGTTCGTGCACGTACTTCGCCGACAAAGCACAGGCTTCCCTCGAGGCAGTTCAAAGTAT
BrAP2a cDNA	(605)	TGACGAATTTAACGAAGGAAGAGTTCGTGCACGTACTTCGCCGACAAAGCACAGGCTTCCCTCGAGGCAGTTCAAAGTAT
BrAP2a DNA	(990)	TGACGAATTTAACGAAGGAAGAGTTCGTGCACGTACTTCGCCGACAAAGCACAGGCTTCCCTCGAGGCAGTTCAAAGTAT
SCM-a cDNA	(724)	TGACGAATTTAACGAAGGAAGAGTTCGTGCACGTACTTCGCCGACAAAGCACAGGCTTCCCTCGAGGCAGTTCAAAGTAT
SCM-a DNA	(1281)	TGACGAATTTAACGAAGGAAGAGTTCGTGCACGTACTTCGCCGACAAAGCACAGGCTTCCCTCGAGGCAGTTCAAAGTAT
		1361 … 1440
Bra011741 cDNA	(685)	AGAGGTGTCACTTTGCATAAGTGTGGTCGTTGGGAAGCTCGAATGGGTCAATTCTTAGGCAAAAAGTA------------
Bra011741 DNA	(1070)	AGAGGTGTCACTTTGCATAAGTGTGGTCGTTGGGAAGCTCGAATGGGTCAATTCTTAGGCAAAAAGTACTTTCATTCATT
BrAP2a cDNA	(685)	AGAGGTGTCACTTTGCATAAGTGTGGTCGTTGGGAAGCTCGAATGGGTCAATTCTTAGGCAAAAAGTA------------
BrAP2a DNA	(1070)	AGAGGTGTCACTTTGCATAAGTGTGGTCGTTGGGAAGCTCGAATGGGTCAATTCTTAGGCAAAAAGTACTTTCATTCATT
SCM-a cDNA	(804)	AGAGGTGTCACTTTGCATAAGTGTGGTCGTTGGGAAGCTCGAATGGGTCAATTCTTAGGCAAAAAGTA------------
SCM-a DNA	(1361)	AGAGGTGTCACTTTGCATAAGTGTGGTCGTTGGGAAGCTCGAATGGGTCAATTCTTAGGCAAAAAGTACTTTCATTCATT
		1441 … 1520
Bra011741 cDNA	(753)	---TGTTTATTT
Bra011741 DNA	(1150)	CATTCTTTATTTTTCTAATAGATTTGTTATTGTAACTTGAGTTTTGATGCTTTAACTTTTCATATTAGGTATGTTTATTT
BrAP2a cDNA	(753)	---TGTTTATTT
BrAP2a DNA	(1150)	CATTCTTTATTTTTCTAATAGATTTGTTATTGTAACTTGAGTTTTGATGCGTTAACTTTTCATATTAGGTATGTTTATTT
SCM-a cDNA	(872)	---TGTTTATTT
SCM-a DNA	(1441)	CATTCTTTATTTTTCTAATAGATTTGTTATTGTAACTTGAGTTTTGATGCGTTAACTTTTCATATTAGGTATGTTTATTT
		1521 … 1600
Bra011741 cDNA	(762)	GGGTTTGTTCGACACCGAGGTTGAAGCTGCTAG---
Bra011741 DNA	(1230)	GGGTTTGTTCGACACCGAGGTTGAAGCTGCTAGGTAAATGTCTTTATGATTGATTCCACAACACACATTGTTAGAAAGCT
BrAP2a cDNA	(762)	GGGTTTGTTCGACACCGAGGTTGAAGCTGCTAG---
BrAP2a DNA	(1230)	GGGTTTGTTCGACACCGAGGTTGAAGCTGCTAGGTAAATGTCTTTCTGATTGATTCCACAACACACATTGTTAGAAAGCT
SCM-a cDNA	(881)	GGGTTTGTTCGACACCGAGGTTGAAGCTGCTAG---
SCM-a DNA	(1521)	GGGTTTGTTCGACACCGAGGTTGAAGCTGCTAGGTAAATGTCTTTCTGATTGATTCCACAACACACATTGTTAGAAAGCT
		1601 … 1680
Bra011741 cDNA	(795)	--AGCTTACGATAAAGCTGCAATCAAATGTAATGGCAA
Bra011741 DNA	(1310)	TTAATCTCGTATAATGTTTTTATTTTATTTTTTAATGACCACAGAGCTTACGATAAAGCTGCAATCAAATGTAATGGCAA
BrAP2a cDNA	(795)	--AGCTTACGATAAAGCTGCAATCAAATGTAATGGCAA
BrAP2a DNA	(1310)	TTAATCTCGTATAATGTTTTTATTTTGTTTTTTGATGACCACAGAGCTTACGATAAAGCTGCAATCAAATGTAATGGCAA
SCM-a cDNA	(914)	--AGCTTACGATAAAGCTGCAATCAAATGTAATGGCAA
SCM-a DNA	(1601)	TTAATCTCGTATAATGTTTTTATTTTGTTTTTTGATGACCACAGAGCTTACGATAAAGCTGCAATCAAATGTAATGGCAA

```
                               1681                                                                          1760
Bra011741 cDNA     (831)   AGACGCTGTGACTAACTTTGATCCAAGCATATACGACGACGAATTGAATGCCG---------------------------
Bra011741 DNA     (1390)   AGACGCTGTGACTAACTTTGATCCAAGCATATACGACGACGAATTGAATGCCGGTAATTTTATTTCAAAATCTCTTTTG
BrAP2a cDNA        (831)   AGACGCTGTGACTAACTTTGATCCAAGCATATACGACGACGAATTGAATGCCG---------------------------
BrAP2a DNA       (1390)   AGACGCTGTGACTAACTTTGATCCAAGCATATACGACGACGAATTGAATGCCGGTAATTTTATTTCAAAATCTTTTTTG
SCM-a cDNA         (950)   AGACGCTGTGACTAACTTTGATCCAAGCATATACGACGACGAATTGAATGCCG---------------------------
SCM-a DNA         (1681)   AGACGCTGTGACTAACTTTGATCCAAGCATATACGACGACGAATTGAATGCCGGTAATTTTATTTCAAAATCTTTTTTG
                               1761                                                                          1840
Bra011741 cDNA     (884)   -----------------------------------------------------------------AGTCATCAGGGAATC
Bra011741 DNA     (1470)   AGTTTTTAATCGTATATTTATGGGTAAGAACTTGGTTTCAAGATTTCTTTTTAAATTTGTGTAGAGTCATCAGGGAATC
BrAP2a cDNA        (884)   -----------------------------------------------------------------AGTCATCAGGGAATC
BrAP2a DNA        (1470)   AGTTTTTAATCGTATCTTTTTGGGAAAGAACTTGGTTTCAAGATTTCTTTTTAAATTTGTGTAGAGTCATCAGGGAATC
SCM-a cDNA        (1003)   -----------------------------------------------------------------AGTCATCAGGGAATC
SCM-a DNA         (1761)   AGTTTTTAATCGTATCTTTTTGGGAAAGAACTTGGTTTCAAGATTTCTTTTTAAATTTGTGTAGAGTCATCAGGGAATC
                               1841                                                                          1920
Bra011741 cDNA     (899)   CTATTCAACAAGATCATAACCTCGATTTGAGTTTGGGAAACTCGGTTAATTCGAAGCAAAAGGGTCAAGATATGCGGCTC
Bra011741 DNA     (1550)   CTATTCAACAAGATCATAACCTCGATTTGAGTTTGGGAAACTCGGTTAATTCGAAGCAAAAGGGTCAAGATATGCGGCTC
BrAP2a cDNA        (899)   CTATTCAACAAGATCATAACCTCGATTTGAGTTTGGGAAACTCGGTTAATTCGAAGCAAAAGGGTCAAGATATGCGGCTC
BrAP2a DNA        (1550)   CTATTCAACAAGATCATAACCTCGATTTGAGTTTGGGAAACTCGGTTAATTCGAAGCAAAAGGGTCAAGATATGCGGCTC
SCM-a cDNA        (1018)   CTATTCAACAAGATCATAACCTCGATTTGAGTTTGGGAAACTCGGTTAATTCGAAGCAAAAGGGTCAAGATATGCGGCTC
SCM-a DNA         (1841)   CTATTCAACAAGATCATAACCTCGATTTGAGTTTGGGAAACTCGGTTAATTCGAAGCAAAAGGGTCAAGATATGCGGCTC
                               1921                                                                          2000
Bra011741 cDNA     (979)   AAG-----------------------------------------------------------------------------
Bra011741 DNA     (1630)   AAGGTAAAGTGTTTATTAAATTATATACTATAGTTTCTACCTTAAATATCTTCGTTTGTTATAAACATTTTTTTCTTTTT
BrAP2a cDNA        (979)   AAG-----------------------------------------------------------------------------
BrAP2a DNA        (1630)   AAGGTAAAGTGTTTATTAAATTATATACTATAGTTTCTACCTTAAATTTCTTCGTTTGTTATAAACATTTTTTTCTTTTT
SCM-a cDNA        (1098)   AAG-----------------------------------------------------------------------------
SCM-a DNA         (1921)   AAGGTAAAGTGTTTATTAAATTATATACTATAGTTTCTACCTTAAATTTCTTCGTTTGTTATAAACATTTTTTTCTTTTT
                               2001                                                                          2080
Bra011741 cDNA     (982)   -----ATGAACCAACAAGATTCTCTTCATCCTAATGAGATTCTTGGATTAGGTCAAACCGGAATGGTTAACCATATCCCA
Bra011741 DNA     (1710)   ATTAGATGAACCAACAAGATTCTCTTCATCCTAATGAGATTCTTGGATTAGGTCAAACCGGAATGGTTAACCATATCCCA
BrAP2a cDNA        (982)   -----ATGAACCAACAAGATTCTCTTCATCCTAATGAGATTCTTGGATTGGGTCAAACCGGAATGGTTAACCATATCCCA
BrAP2a DNA        (1710)   ATTAGATGAACCAACAAGATTCTCTTCATCCTAATGAGATTCTTGGATTGGGTCAAACCGGAATGGTTAACCATATCCCA
SCM-a cDNA        (1101)   -----ATGAACCAACAAGATTCTCTTCATCCTAATGAGATTCTTGGATTGGGTCAAACCGGAATGGTTAACCATATCCCA
SCM-a DNA         (2001)   ATTAGATGAACCAACAAGATTCTCTTCATCCTAATGAGATTCTTGGATTGGGTCAAACCGGAATGGTTAACCATATCCCA
                               2081                                                                          2160
Bra011741 cDNA    (1057)   AATTCAAATCTCCAA-----------------------------------------------------------------
Bra011741 DNA     (1790)   AATTCAAATCTCCAAGTGAGTAAAAA---CACACAAGAAGATATAAAACAATTGTTTAAATTTCAATCGGTTATATTTTT
BrAP2a cDNA       (1057)   AATTCAAATCTCCAA-----------------------------------------------------------------
BrAP2a DNA        (1790)   AATTCAAATCTCCAAGTGAGTAAAAAACACACACAAGAAGATATAAAACATTTGTTTAAATTTCTATCGGTTATGTTTTT
SCM-a cDNA        (1176)   AATTCAAATCTCCAA-----------------------------------------------------------------
SCM-a DNA         (2081)   AATTCAAATCTCCAAGTGAGTAAAAAACACACACAAGAAGATATAAAACATTTGTTTAAATTTCTATCGGTTATGTTTTT
                               2161                                                                          2240
Bra011741 cDNA    (1072)   --------------------------------------------TTTCCGGGCAGCAGCAACATTGGTGGCGGAGGAGGA
Bra011741 DNA     (1867)   ACTAACAGAATACAGTATGTATATTTTTGTATGGTGTAAATTAGTTTCCGGGCAGCAGCAACATTGGTGGCGGAGGAGGA
BrAP2a cDNA       (1072)   --------------------------------------------TTTCGGGGCAGCAGCAACATTGGTGGCGGAGGAGGA
BrAP2a DNA        (1870)   ACTAA-AGAATA-----TGTATATTTTTGTATGGTGTAAATTAGTTTCGGGGCAGCAGCAACATTGGTGGCGGAGGAGGA
SCM-a cDNA        (1191)   --------------------------------------------TTTCGGGGCAGCAGCAACATTGGTGGCGGAGGAGGA
SCM-a DNA         (2161)   ACTAA-AGAATA-----TGTATATTTTTGTATGGTGTAAATTAGTTTCGGGGCAGCAGCAACATTGGTGGCGGAGGAGGA
                               2241                                                                          2320
Bra011741 cDNA    (1108)   TTCTCACTATTTCCGGTGGCTGAGAACCACCGGTTTGATGGTCGGACCACGACGAACCAAGTGTTGGCAAATGCTGCAGC
Bra011741 DNA     (1947)   TTCTCACTATTTCCGGTGGCTGAGAACCACCGGTTTGATGGTCGGACCACGACGAACCAAGTGTTGGCAAATGCTGCAGC
BrAP2a cDNA       (1108)   TTCTCACTATTTCCGGTGGCTGAGAACCACCGGTTTGATGGTCGGACCACGACGAACCAAGTGTTGGCAAATGCTGCAGC
BrAP2a DNA        (1944)   TTCTCACTATTTCCGGTGGCTGAGAACCACCGGTTTGATGGTCGGACCACGACGAACCAAGTGTTGGCAAATGCTGCAGC
SCM-a cDNA        (1227)   TTCTCACTATTTCCGGTGGCTGAGAACCACCGGTTTGATGGTCGGACCACGACGAACCAAGTGTTGGCAAATGCTGCAGC
SCM-a DNA         (2235)   TTCTCACTATTTCCGGTGGCTGAGAACCACCGGTTTGATGGTCGGACCACGACGAACCAAGTGTTGGCAAATGCTGCAGC
                               2321                                                                          2400
Bra011741 cDNA    (1188)   ATCATCAGGATTCTCTCCTCATCATCACAATCAGATTTTTAATTCCACTTCTACTTCTCATCAAAATTGGCTGCAGACCA
Bra011741 DNA     (2027)   ATCATCAGGATTCTCTCCTCATCATCACAATCAGATTTTTAATTCCACTTCTACTTCTCATCAAAATTGGCTGCAGACCA
BrAP2a cDNA       (1188)   ATCATCAGGATTCTCTCCTCATCATCACAATCAGATTTTTAATTCCACTTCTACTTCTCATCAAAATTGGCTGCAGACCA
BrAP2a DNA        (2024)   ATCATCAGGATTCTCTCCTCATCATCACAATCAGATTTTTAATTCCACTTCTACTTCTCATCAAAATTGGCTGCAGACCA
SCM-a cDNA        (1307)   ATCATCAGGATTCTCTCCTCATCATCACAATCAGATTTTTAATTCCACTTCTACTTCTCATCAAAATTGGCTGCAGACCA
SCM-a DNA         (2315)   ATCATCAGGATTCTCTCCTCATCATCACAATCAGATTTTTAATTCCACTTCTACTTCTCATCAAAATTGGCTGCAGACCA
                               2401                              2435
Bra011741 cDNA    (1268)   ATGGCTTCCAACCTTCTCTCATGAGACCTTCTTGA
Bra011741 DNA     (2107)   ATGGCTTCCAACCTTCTCTCATGAGACCTTCTTGA
BrAP2a cDNA       (1268)   ATGGCTTCCAACCTTCTCTCATGAGACCTTCTTGA
BrAP2a DNA        (2104)   ATGGCTTCCAACCTTCTCTCATGAGACCTTCTTGA
SCM-a cDNA        (1387)   ATGGCTTCCAACCTTCTCTCATGAGACCTTCTTGA
SCM-a DNA         (2395)   ATGGCTTCCAACCTTCTCTCATGAGACCTTCTTGA
```

Note: *BrAP2a* is an *AP2* gene derived from wild type *B. rapa* by our PCR amplification. *Bra011741* is the *BrAP2a* gene from *Brassica rapa* database. The *SCM-a* is the *BrAP2a* gene in the sepal carpeloid mutant *scm*. Red highlighted domain showed DNA repeat sequence in the *SCM-a*, and green highlighted domain showed cDNA repeat sequence in the *SCM-a*.
