# Supplementary material for: Defective APETALA2 Genes Lead to Sepal Modification in Brassica Crops
Source: Front Plant Sci. 2018 Mar 20;9:367. doi: 10.3389/fpls.2018.00367 (PMC5869249; doi:10.3389/fpls.2018.00367)
Supplement: Supplementary file 4 [file Image_3.PDF]

Supplemental Figure 3

Alignments of AP2 proteins between the SCM mutant and wild type *B.rapa*

```
                1                                                                        75
AtAP2      (1)  MWDLNDAPHQTQREEESEEFCYSSPSKRVGSFSNSSSSAVVIEDGSDDDELNRVRPNNPLVTHQFFPEMDS----
Bol028934  (1)  MWDLNDSPHQTLVEEESEELCYSSPGKRVGSFSNSSSSAVVIEDGSDDDEPNRVRPNNPLVTHQFFPEMETSVGD
Bra011741  (1)  MWDLNDSPHQTLVEEESEELCYSSPGKRVGSFSNSSSSAVVIEDGSDDDEHNRVRPNNQLVTHQFFPQMETSVGD
Brp.AP2.a  (1)  MWDLNDSPHQTLVEEESEELCYSSPGKRVGSFSNSSSSAVVIEDGSDDDEPNRVRPNNPLVTHQFFPQMETSVGD
Brp.AP2.b  (1)  MWDLNDSPHQTVRQEESEEFCYSSPGKRVGSFSTSSSSAVVIEDGSDDDEPNRVRPNNPLITHQFFPEMESSVGD
Bra017809  (1)  MWDLNDSPHQTVRQEESEEFCYSSPGKRVGSFSTSSSSAVVIEDGSDDDEPNRVRPNNPLITHQFFPEMESSVGD
SCM-b      (1)  MWDLNDSPHQTVRQEESDEFCYSSPGKRVGSFSTSSSSAVVIEDGSDDDEPNRVRPNNPLITHQFFPEMESSVGD
Bol018627  (1)  MWDLNDSPHQTVRQEESEEFCYSSPGKRVGSFSNSSSSAVVIEDGSDDDEPNRVRANNPLITHQFFPEMESSVGD
                76                                                                      150
AtAP2     (72)  NG-GGVASGFPRAHWFGVKFCQSDLATGSSAGKATNVAAAVVEPAQPLKKSRRGPRSRSSQYRGVTFYRRTGRWE
Bol028934 (76)  DGGGGPGSGFPRSHWFGVKFCQSDLATGSSAGKPATVA-AVVEPAQPLKKSRRGPRSRSSQYRGVTFYRRTGRWE
Bra011741 (76)  DGGGGPGSGFPRSHWFGVKFCQSDLATGSSAGKPATVA-PVVEPAQPLKKSRRGPRSRSSQYRGVTFYRRTGRWE
Brp.AP2.a (76)  DGGGGPGSGFPRSHWFGVKFCQSDLATGSSAGKPATVA-PVAEPAQPLKKSRRGPRSRSSQYRGVTFYRRTGRWE
Brp.AP2.b (76)  NGGGGTGPGFPRSHWFGVQFSQSDLATGSSVGKPATVG-AVVEPTQPLKKSRRGPRSRSSQYRGVTFYRRTGRWE
Bra017809 (76)  NGGGGTGPGFPRSHWFGVQFSQSDLATGSSVGKPATVG-AVVEPTQPLKKSRRGPRSRSSQYRGVTFYRRTGRWE
SCM-b     (76)  NGGGGTGPGFPRSHWFGVQFSQSDLATGSSVGKPATVG-AVVEPTQPLKKSRRGPRSRSSQYRGVTFYRRTGRWE
Bol018627 (76)  NGGGGPGSSFPRSHWFGVQFSQSDLATGSSVGKPATVG-AVVEPAQPLKKSRRGPRSRSSQYRGVTFYRRTGRWE
                151                                                                     225
AtAP2    (146)  SHIWDCGKQVYLGGFDTAHAAARAYDRAAIKFRGVEADINFNIDDYDDDLKQMTNLTKEEFVHVLRRQSTGFPRG
Bol028934 (150) SHIWDCGKQVYLGGFDTAHAAARAYDRAAIKFRGVEADINFTIEDYDDDLKQMTNLTKEEFVHVLRRQSTGFPRG
Bra011741 (150) SHIWDCGKQVYLGGFDTAHAAARAYDRAAIKFRGVEADINFTIEDYDDDLKQMTNLTKEEFVHVLRRQSTGFPRG
Brp.AP2.a (150) SHIWDCGKQVYLGGFDTAHAAARAYDRAAIKFRGVEADINFTIEDYDDDLKQMTNLTKEEFVHVLRRQSTGFPRG
Brp.AP2.b (150) SHIWDCGKQVYLGGFDTAHAAARAYDRAAIKFRGVEADINFNIEDYDNDMKQMTNLTKEEFVHVLRRQSTGFPRG
Bra017809 (150) SHIWDCGKQVYLGGFDTAHAAARAYDRAAIKFRGVEADINFNIEDYDNDMKQMTNLTKEEFVHVLRRQSTGFPRG
SCM-b    (150)  SHIWDCGKQVYLGGFDTAHAAARAYDRAAIKFRGVEADINFNIEDYDNDMKQMTNLTKEEFVHVLRRQSTGFPRG
Bol018627 (150) SHIWDCGKQVYLGGFDTAHAAARAYDRAAIKFRGVEADINFNIEDYDNDMKQMTNLTKEEFVHVLRRQSTGFPRG
                226                                                                     300
AtAP2    (221)  SSKYRGVTLHKCGRWEARMGQFLGKKYVYLGLFDTEVEAARAYDKAAIKCNGKDAVTNFDPSIYDEELNAESSGN
Bol028934 (225) SSKYRGVTLHKCGRWEARMGQFLGKKYVYLGLFDTEVEAARAYDKAAIKCNGKDAVTNFDPSIYDDELNAESSGN
Bra011741 (225) SSKYRGVTLHKCGRWEARMGQFLGKKYVYLGLFDTEVEAARAYDKAAIKCNGKDAVTNFDPSIYDDELNAESSGN
Brp.AP2.a (225) SSKYRGVTLHKCGRWEARMGQFLGKKYVYLGLFDTEVEAARAYDKAAIKCNGKDAVTNFDPSIYDDELNAESSGN
Brp.AP2.b (225) SSKYRGVTLHKCGRWEARMGQFLGKKYVYLGLFDTEVEAARAYDKAAIKCNGKDAVTNFDSSIYDEELNAESSGN
Bra017809 (225) SSKYRGVTLHKCGRWEARMGQFLGKKYVYLGLFDTEVEAARAYDKAAIKCNGKDAVTNFDSSIYDEELNAESSGN
SCM-b    (225)  SSKYRGVTLHKCGRWEARMGQFLGKKYVYLGLFDTEVEAARAYDKAAIKCNGKDAVTNFDSSIYDEELNAESSGN
Bol018627 (225) SSKYRGVTLHKCGRWEARMGQFLGKKYVYLGLFDTEVEAARAYDKAAIKCNGKDAVTNFDSSIYDEELNAESSGN
                301                                                                     375
AtAP2    (296)  PTTPQDHNLDLSLGNSANSKHKSQDMRLRMNQQQQDSLHSNEVLGLGQTGMLNHTPNSNHQFPGSSNIGSGGGFS
Bol028934 (300) -SIQQDHNLDLSLGNSVNSKQKGQDMRLKMNQQ--ETLHPNEILGLGQTGMVNHIPNSNLQFPGSSNIGGGGGFS
Bra011741 (300) -PIQQDHNLDLSLGNSVNSKQKGQDMRLKMNQQ--DSLHPNEILGLGQTGMVNHIPNSNLQFPGSSNIGGGGGFS
Brp.AP2.a (300) -PIQQDHNLDLSLGNSVNSKQKGQDMRLKMNQQ--DSLHPNEILGLGQTGMVNHIPNSNLQFRGSSNIGGGGGFS
Brp.AP2.b (300) -PIQQDHNLDLSLGNSVNSKHKGQDMRLKMNQQ--DSLHSNEILGLGQTGMVNYIPNSVHQFPGSSNIHRGGGFS
Bra017809 (300) -PIQQDHNLDLSLGNSVNSKHKGQDMRLKMNQQ--DSLHSNEILGLGQTGMVNYIPNSVHQFPGSSNIHRGGGFS
SCM-b    (300)  -PIQQDHNLDLSLGNSVNSKHKGQDMRLKMNQQ--DSLHSNEILGLGQTGMVNYIPNSVHQFPGSSNIHRGGGFS
Bol018627 (300) -PIQHDHNLDLSLGNSVNSKHNGQDMRLKMNQQ--DSLHSNEILGLGP-GMVNYIPDSVHQFPGSSNIHGGGGFS
                376                                                          437
AtAP2    (371)  LFPAAENHRFDGRASTNQVLTNAAASSGFSPHHHNQIFNSTSTPHQNWLQTNGFQPPLMRPS
Bol028934 (372) LFPVAENHRFDGRTTTNQVLANAAASSGFSPHHHNQIFNSTSTSHQNWLQTNGFQPS-----
Bra011741 (372) LFPVAENHRFDGRTTTNQVLANAAASSGFSPHHHNQIFNSTSTSHQNWLQTNGFQPSLMRPS
Brp.AP2.a (372) LFPVAENHRFDGRTTTNQVLANAAASSGFSPHHHNQIFNSTSTSHQNWLQTNGFQPSLMRPS
Brp.AP2.b (372) LFPVTENHQFEGRTTANQVLANAAASSGFSPHNHNQIFNSTPTSHQNWLQTNGFQPPLMRP-
Bra017809 (372) LFPVTENHQFEGRTTANQVLANAAASSGFSPHNHNQIFNSTPTSHQNWLQTNGFQPPLMRPS
SCM-b    (372)  LFPVTENHQFEGRTTANQVLANAAASSGFSPHNHNQIFNSTPTSHQNWLQTNGFQPPLMRP-
Bol018627 (371) LFPVTENHQFEGRTTANQVLANAAASSGFSPHNHHQIFNSTPTSHQNWLQTNGFQPPLMRP-
```

Note: Bra017809 and Bra011741 are two AP2 proteins of *B.rapa* from *B. rapa* database. Bol018627 and Bol028934 are two AP2 proteins of *B.oleracea* from *B. rapa* database. Brp.AP2.a and Brp.AP2.b are two *B. rapa* AP2 proteins derived from our PCR amplification. AtAP2 is AP2 protein of *Arabidopsis*.
