# Supplementary material for: Defective APETALA2 Genes Lead to Sepal Modification in Brassica Crops
Source: Front Plant Sci. 2018 Mar 20;9:367. doi: 10.3389/fpls.2018.00367 (PMC5869249; doi:10.3389/fpls.2018.00367)
Supplement: Supplementary file 5 [file Image_4.PDF]

Supplemental Figure 4

The constructs for *B. napus* transformation and sequencing results of *ap2* quadruple mutant

A

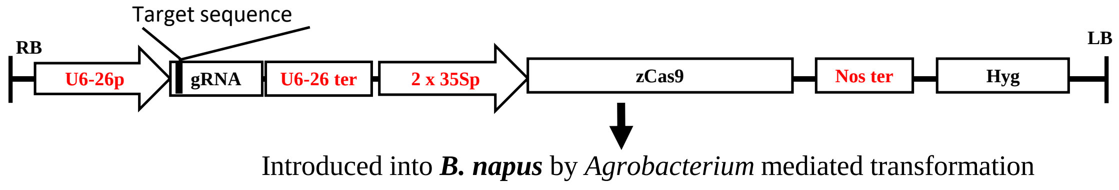

B

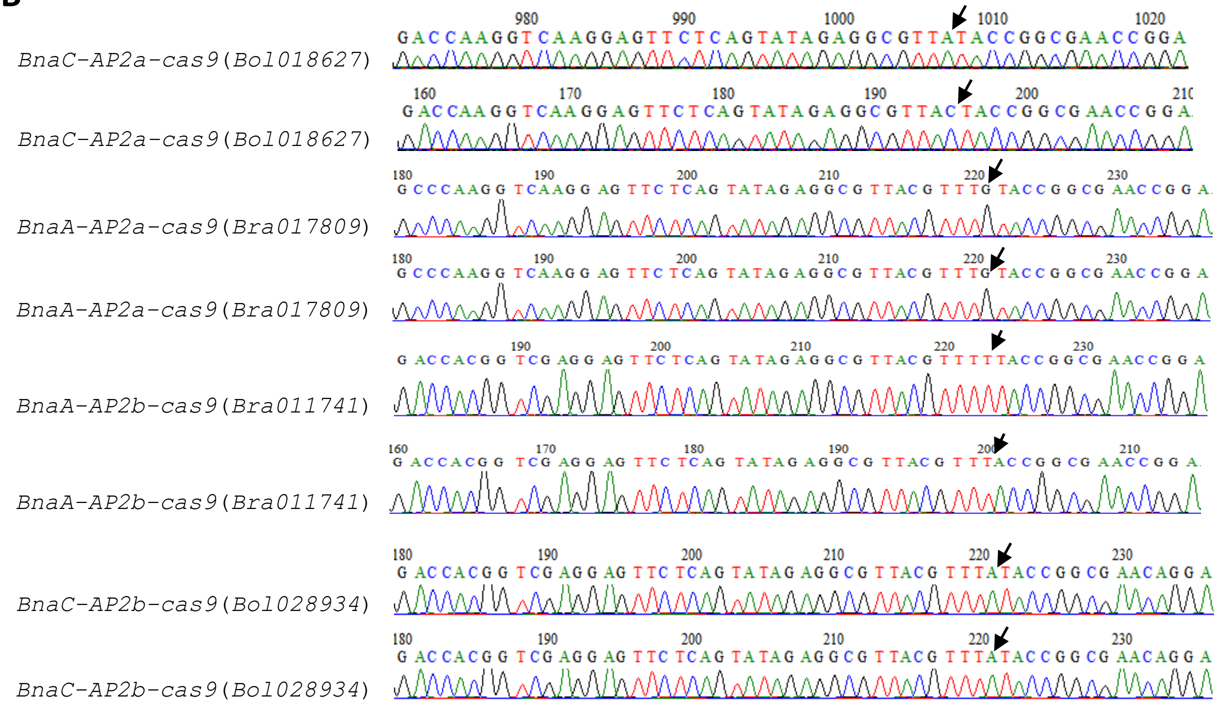

Note: A, Constructs used for *B. napus* transformation mediated by *Agrobacterium.* U6-26p:*Arabidopsis* U6 gene promoter; U6-26 ter, U6-26 terminator; 2x35Sp: 2x35S promoter; Nos ter: Nos terminator; RB: right border; LB: left border; gRNA: single guide RNA with the target sequence; zCAS9: codon-optimized Cas9 gene from *Zea mays*; Hyg: hygromycin. B, Sequencing chromatogram of *ap2* corresponding to Figure 5C. The arrow bar indicates the mutation sites.
